# Why Is Working Memory Performance Unstable? A Review of 21 Factors

**DOI:** 10.5964/ejop.v14i1.1472

**Published:** 2018-03-12

**Authors:** Rachael N. Blasiman, Christopher A. Was

**Affiliations:** a Kent State University, Kent, OH, USA; Department of Psychology, Webster University Geneva, Geneva, Switzerland; University of Bari "A. Moro", Bari, Italy

**Keywords:** working memory, individual differences

## Abstract

In this paper, we systematically reviewed twenty-one factors that have been shown to either vary with or influence performance on working memory (WM) tasks. Specifically, we review previous work on the influence of intelligence, gender, age, personality, mental illnesses/medical conditions, dieting, craving, stress/anxiety, emotion/motivation, stereotype threat, temperature, mindfulness training, practice, bilingualism, musical training, altitude/hypoxia, sleep, exercise, diet, psychoactive substances, and brain stimulation on WM performance. In addition to a review of the literature, we suggest several frameworks for classifying these factors, identify shared mechanisms between several variables, and suggest areas requiring further investigation. This review critically examines the breadth of research investigating WM while synthesizing the results across related subfields in psychology.

Working memory (WM) is an individual’s capacity to simultaneously manipulate some information while maintaining other information (Baddeley, 1986; Kane et al., 2004; Miyake, 2001). Many researchers view the measurement of WM as a straightforward task – a variety of WM tests are available, and all that is necessary is to select a measure or two, administer them to participants, and assign participants a numerical result. The resulting value can then be used to account for variance in other cognitive variables, to divide participants into high and low WM groups, to examine changes over time, or to serve as a baseline comparison or covariate for interventions or experimental treatments. Regardless of the study design, the measurement of WM is frequently treated as a relatively stable, valid, descriptor of a person’s true WM capacity. However, our review of the broader literature indicates that performance on WM tasks is sensitive to many variables and situational factors. Because of the burgeoning numbers of researchers who investigate WM or who include it as a measure of cognitive function, we feel it is important to consider the surprising number of factors that can influence the measurement of WM and how these factors may influence the interpretation of results. In that light, this paper serves three important functions.

First, this review should be viewed as a resource. We conducted an exhaustive search of the literature to identify articles that met the broad requirements of this review; namely, we review articles that included any factor that can alter WM performance. Out of the several thousand articles examined, several hundred were related to the goals for this paper. Representative articles for each factor were selected and incorporated into the review, and a supplement is available in which all reviewed articles are listed and classified by topic. As a resource, this paper highlights and describes each factor and relevant research findings. This information would be helpful for researchers just entering the field and also serves as a broad overview for more experienced WM researchers. Second, we present a framework that classifies and groups these factors in a meaningful way. This framework allows for the classification of different types of factors, which in turn will promote discussion as to the manner in which they impact WM performance and will foster the development of cross-disciplinary hypotheses on the underlying mechanisms that cause variability in WM performance. We present additional frameworks in the conclusion of this paper. Third, we hope that this paper provides a comprehensive overview of factors that can influence WM research, so that gaps or omissions in the literature can be identified and new lines of inquiry can form. To that end, we conclude this review with recommendations for future research in these areas.

Table 1 lists the twenty-one factors that will be reviewed in this paper and attempts to categorize them on several dimensions. The first dimension, and the one that provides the framework for this paper, divides factors into two groups: individual differences factors and manipulated (or environmental) factors. Manipulated factors are more malleable, often temporary or reversible in nature, and are generally ones that can be randomly assigned to a person or obtained though some intervention. Of course, these factors can be categorized in many ways (see Table 1), and we will consider several of these classification systems in the conclusion to this paper. Several of the possible categorization systems are listed in Table 1, such as length of effect, malleability, and presumed mechanism. However, many gaps and inconsistencies exist in the literature, and this table only reflects an overall assessment based on the literature reviewed in this paper.

**Table 1:** A Summary of Factors That Influence WM

Factors	Acute vs Chronic effect	Malleability Low/Med/High	Mechanism/Presumed cause of effect	Consistency/ Inconsistency in literature
Individual Differences				
Intelligence	C	L	Biological	C
Gender	C	L	Biological	I
Age	C	L	Biological	C
Personality	C	L	Biological	I
Mental illness / Medical conditions	C	M	Preoccupying thoughts/Biological	C
Manipulated				
Emotion	A	M	Preoccupying thoughts	C
Stress/Anxiety	A	M	Preoccupying thoughts	C
Dieting	A/C^b^	H	Preoccupying thoughts	C
Craving	A	H	Preoccupying thoughts	I
Stereotype threat	A	H	Preoccupying thoughts	C
Temperature	A	H	Preoccupying thoughts/Biological	Insufficient
Mindfulness	Uncertain	H	Preoccupying thought control	Insufficient
Practice	Uncertain	H	Uncertain	I
Sleep	A	H	Uncertain	C
Bilingualism	C	M	Uncertain	C
Musical training	C	M	Uncertain	Insufficient
Altitude/Hypoxia	A/C*	H	Biological	Insufficient
Exercise	A/C*	H	Biological	I
Diet	A/C*	H	Biological	Insufficient
Drug Use	A/C*	H	Biological	C
Brain stimulation	A	H	Biological	C

^a^May be either an acute or chronic effect, depending on circumstances.

Before reviewing the individual factors, a few remarks about WM are needed to provide a theoretical base and to describe some terms that will be used throughout the text. First, we have aligned this review with the theoretical model of WM initially proposed by Baddeley and Hitch (1974). According to this model (Figure 1), WM consists of multiple specialized components that allow humans to comprehend and interact with the environment, represent and retain information about the immediate past, and act on certain goals.

The three major components of the original model are the central executive, the phonological loop, and the visual-spatial sketchpad. The phonological loop and the visual-spatial sketchpad maintain memory traces that overlap with sensory memory using rehearsal in the phonological loop and image generation in the visual-spatial sketchpad. The central executive coordinates these two memory systems and controls attention, focus, and attention switching, as well as the activation of long-term memory. The phonological loop is fractionated into a passive phonological store and an active rehearsal the process. The phonological store represents material in a phonological code that decays with time unless the material is rehearsed. The visual-spatial sketchpad is also fractionated into passive and active subsystems. Logie (1999) refers to these as the passive visual cache and an active inner-scribe. In more recent versions of the multiple components model, Baddeley (2000; Baddeley & Logie, 1999) includes an episodic buffer. The episodic buffer is a storage system that uses multimodal elements to coordinate the memory systems with long-term memory. This leaves the central executive as an attention system not limited to WM processes.

Certain factors discussed in this paper may selectively target the central executive, phonological loop, visuospatial sketchpad, or the episodic buffer; these are noted where appropriate. In all other cases, the impairment is to WM performance in general. This paper generally operates within the framework of Baddeley’s (1986) model of WM, and these terms are used throughout the text. Other theoretical models of WM have been proposed (e.g. Cowan, 1999) and many definitions provided (see Cowan, 2017); however, a thorough review of theory is outside the scope of this work.

**Figure 1:**
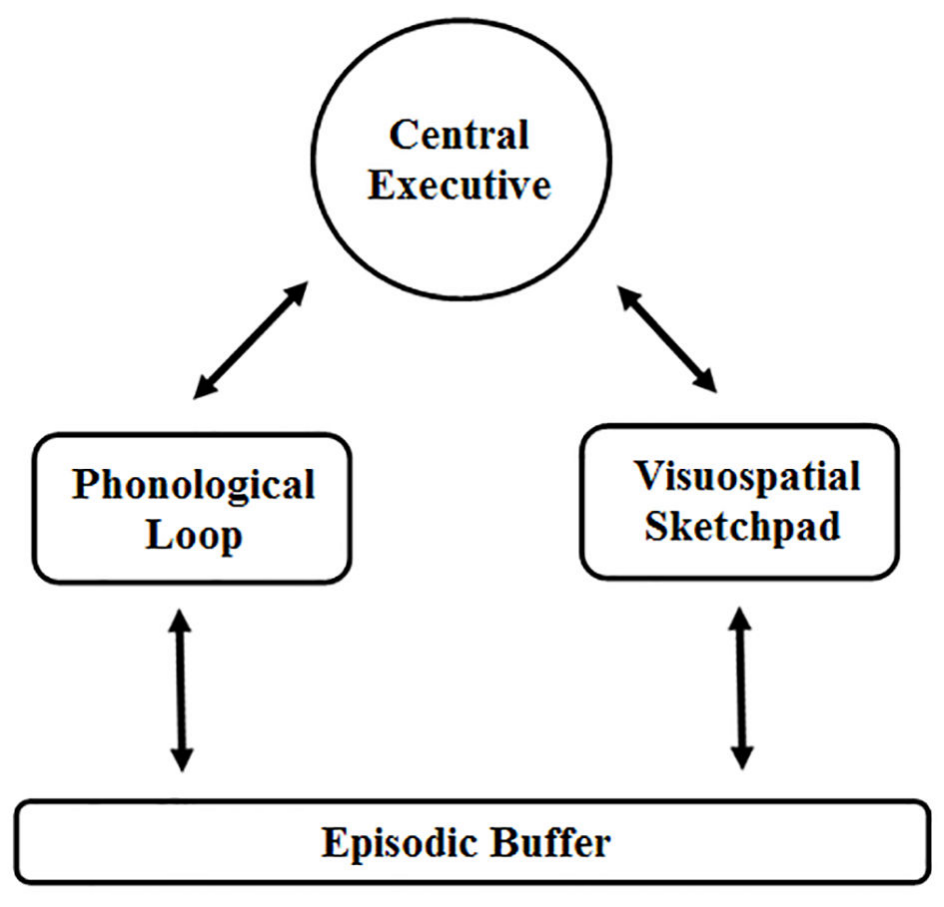
Basic theoretical framework for this review (based on Baddeley, 1986).

Second, we frequently reference two commonly used classes of WM tasks: n-back tasks and complex span tasks. An n-back task presents a participant with a series of stimuli, such as objects or letters of the alphabet. The participant must make a decision on every stimulus, and determine if it is the same one that was presented *n* stimuli ago. Often a 2-back task is selected, which is generally considered a low load task, but task difficulty can be increased by increasing the number of objects that a person must maintain and update within the paradigm. The second class of WM tasks is complex span tasks, such as the reading span (RSPAN, Daneman & Carpenter, 1980) or operation span (OSPAN; Turner & Engle, 1989). These types of tasks generally have two goals that must be simultaneously achieved: (1) to maintain information, such as a word or letter of the alphabet and (2) to actively manipulate other information, such as solving math problems or judging the veracity of sentences. Finally, many different variations of WM tasks have been employed to assess WM, as these tests can use either verbal or spatial information, and a wide variety of stimuli may be employed within the task. The wide variety of tasks, stimuli, and load create challenges in interpreting or comparing results of WM studies.

Although n-back tasks are often used to measure WM, they are not without concerns. Kane, Conway, Miura, and Colflesh (2007) found that although n-back tasks may have face validity as a WM measure, they did not show convergent validity with a popular complex span task. Jaeggi, Buschkuehl, Perrig, and Meier (2010) concluded that the n-back task is not a valid measure of individual differences in WM. Indeed, Redick and Lindsey (2013) contend that complex span tasks and n-back tasks are not interchangeable as measures of WM. Therefore, we recommend the reader consider the research reviewed carefully and consider the WM tasks used in each of the studies.

## Individual Differences Factors

We begin this review with summaries of research on individual differences factors. These factors all share the common feature of being relatively stable, often pre-determined characteristics, and include intelligence, age, gender, personality, and both mental and medical conditions.

### Intelligence

Most psychologists are in agreement that WM and general intelligence (*g)* are highly related but separate constructs (Colom, Rebollo, Palacios, Juan-Espinosa, & Kyllonen, 2004; Conway, Cowan, Bunting, Therriault, & Minkoff, 2002; Kane et al., 2004). WM has been specifically associated with fluid intelligence (*g*F; novel problem solving) and not crystallized intelligence (*g*C; verbal and learned experience) in many of these studies (Ackerman, Beier, & Boyle, 2005; Conway et al., 2002; Martínez & Colom, 2009; Unsworth & Engle, 2005). Correlations between WM and *g*F differ from study to study, but tend to be moderate to large, ranging from .34 (Engle, Tuholski, Laughlin, & Conway, 1999; Unsworth & Engle, 2005) to .70 (Colom et al., 2004). In their meta-analysis of WM and *g*, Ackerman et al. (2005) report an average correlation of .48 between these variables, and make note of the wide variety of correlations reported by others.

These discrepancies may be due in part to error or varying participant characteristics, but another possibility is that researchers using different types of WM tasks found different relationships to *g*. This is highlighted by some studies that compare different types of WM tasks, such as operation span, reading span, and spatial WM, as well as n-back tasks of WM. For example, Jaeggi, Studer-Luethi, et al. (2010) report a correlation of .44 between an n-back task and a measure of *g*F, but a correlation of .24 was reported between the OSPAN and that same *g*F measure. The relationship between WM and *g* is further complicated by discriminations between spatial and verbal WM and crystallized and fluid *g*. For instance, Engle et al. (1999) found a difference between overall WM and verbal WM, with a correlation of .34 between the OSPAN and *g*, but .28 for the reading span and *g*. Other researchers have specifically claimed that verbal WM is related to *g*C and spatial WM is related to *g*F (Haavisto & Lehto, 2005; Schweizer & Moosbrugger, 2004).

Some investigations of individuals with borderline IQ (i.e. 55-85) have also been conducted (e.g., Van der Molen, Van Luit, Jongmans, & Van der Molen, 2007). Alloway (2010) examined people with IQs between 70-85 and found that both verbal and spatial WM were impaired in this group. Both verbal and spatial WM functioning are impaired in students with learning disabilities as well (Maehler & Schuchart, 2009). In terms of developmental disability, both Down’s syndrome (Numminen, Service, Ahonen, & Ruoppila, 2001) and William’s syndrome (O’Hearn, Courtney, Street, & Landau, 2009) are associated with deficits in WM performance. In particular, the phonological loop is impaired in children with Down syndrome (Bird & Chapman, 1994; Wang & Bellugi, 1994).

Given the voluminous research on the relationship between WM and intelligence, it is impossible to present more than a brief overview in this paper. However, we can conclude that a close positive relationship between these two variables exists, although the two are not isomorphic (Beier & Ackerman, 2005; Conway, Kane, & Engle, 2003). Given the previous discussion, we label intelligence as a chronic effect of biological origin with low malleability and a high degree of consistency in the literature (Table 1).

### Age

Several groups have examined the three-factor model of WM (the central executive, phonological loop, and visuospatial sketchpad; e.g. Baddeley, 1986) across the life span. The three factors appear to emerge around age six (Gathercole, Pickering, Ambridge, & Wearing, 2004). The basic three-factor structure remains throughout the rest of adulthood and into old age (Hale et al., 2011), even as WM decreases throughout.

Many studies have reported age-related declines in WM (Babcock & Salthouse, 1990; Borella, Carretti, & De Beni, 2008; Cowan, Naveh-Benjamin, Kilb, & Saults, 2006; Craik & Bialystok, 2006; Li et al., 2008; McGinnis & Zelinski, 2003; Park et al., 2002; Paxton, Barch, & Racine, 2008; Salthouse, 1994). In a meta-analysis of age and WM, Bopp and Verhaeghen (2007) reported moderate to large negative correlations between WM and age. WM increases linearly up to adolescence, when it begins to level off, but no change in the basic structure of the factors occurs during this process (Lambek & Shevlin, 2011). The decline appears to be linear, with a constant and continuous rate of decline beginning in the twenties and no sharp decline seen in old age (Borella et al., 2008). In addition to group comparisons, longitudinal designs have also been employed and found consistent decline in WM performance with age (Chiappe, Hasher, & Siegel, 2000; Jenkins, Myerson, Hale, & Fry, 1999; Park et al., 2002; Siegel, 1994).

There have been reports that spatial WM declines more with age than verbal WM does (Hale et al., 2011; Jenkins et al., 1999; Myerson, Hale, Rhee, & Jenkins, 1999), although Borella et al. (2008) did not find this in their analysis. Other researchers have reported only decreases in verbal WM (Fastenau, Denburg, & Abeles, 1996; Vecchi, Richardson, & Cavallini, 2005). Still others report declines in both verbal and spatial WM (de Ribaupierre & Lecerf, 2006; Kemps & Newson, 2006; Park et al., 2002; Salthouse, 1995). In addition to spatial/verbal WM tasks, age differences have been documented in WM for objects (Hartley, Speer, Jonides, Reuter-Lorenz, & Smith, 2001), and faces (Grady et al., 1995; Grady et al., 1998). A notable exception to the age-related decline in WM is a study by Mikels, Larkin, Reuter-Lorenz, and Carstensen (2005) in which older adults (ages 64-80) did better on a WM task employing positive emotion stimuli but younger adults (ages 18-28) did better when negative emotion stimuli were used.

Borella et al. (2008) suggest that WM declines are due to the inability to suppress intrusions of past material. Hedden and Park (2001) found older adults showed greater retroactive interference, leading them to surmise that irrelevant information is not efficiently deleted from WM. In contrast to this view, Fisk and Warr (1996) found that controlling for perceptual speed removed all age-related variance in WM. Gaillard, Barrouillet, Jarrold, and Camos (2011) compared 3^rd^ and 6^th^ graders on a complex span task and found that processing speed was the source of variance between the two groups.

Although the debate continues on the cause and rate of change in WM subcomponents over time, it is clear that WM performance does decline with age and hence it is an important individual differences factor to take into account when assessing WM. Therefore, we label this factor in Table 1 as biological, having a chronic effect over time, and low on malleability (as only the passage of time can alter age). Age also plays a moderating role in the effect of several manipulated factors, such as practice, which will be discussed later in this paper.

### Gender

No general consensus in the field exists when it comes to the relationship between gender and WM performance. Several researchers report that men have an advantage on spatial WM tasks and that women have an advantage on verbal WM tasks (Speck et al., 2000; Voyer, Voyer, & Bryden, 1995), some researchers report only a spatial advantage for men (Lejbak, Crossley, & Vrbancic, 2011; Orsini, Simoetta, & Marmorato, 2004), and others report no differences at all between genders (Nagel, Ohannessian, & Cummins, 2007; Robert & Savoie, 2006).

For instance, Schmidt et al. (2009) found no difference in accuracy, speed, or brain activity (using fMRI) between men and women completing a verbal n-back task, and yet multiple researchers have confirmed differences in prefrontal cortex (PFC) activity between men and women while completing WM tasks (Goldstein et al., 2005; Schweinsburg, Nagel, & Tapert, 2005; Speck et al., 2000). Duff and Hampson (2001) attribute the gender difference to the effect of androgen and estrogen on neurons in the dorsolateral PFC (dlPFC). In contrast with other findings, Duff and Hampson (2001) found a response time and accuracy advantage for females in spatial WM but not for verbal WM, even though males outperformed females on the mental rotation task. Interestingly, Keenan, Ezzat, Ginsburg, and Moore (2001) found that an estrogen supplement can increase verbal WM. In a large sample analysis using automated complex span tasks, Redick et al. (2012) found little to no evidence of gender differences.

Because of the great amount of inconsistency in the results, it is difficult to determine the effect, if any, of gender on WM performance. If a difference does exist, this difference would be relatively chronic and most likely biological in origin (see Table 1). However, given the available research, a firm conclusion cannot be drawn.

### Personality

One of the most commonly used personality frameworks is the Big 5, which describes personality using five broad domains: Conscientiousness, Agreeableness, Neuroticism, Openness to Experience, and Extroversion/introversion (Costa & McCrea, 1978). Of these, Extroversion/introversion has been linked with WM performance most frequently. Extroverts are people who enjoy and actively seek out social situations, while introverts are seen as more socially inhibited (Costa & McCrea, 1978). The link between Extroversion/introversion and WM performance suffers from conflicting evidence, however. Some researchers state that extroverts outperform introverts on WM tasks (Lieberman, 2000; Lieberman & Rosenthal, 2001) yet others find no differences (Fink, Grabner, Neuper, & Neubauer, 2005; Studer-Luethi, Jaeggi, Buschkuehl, & Perrig, 2012).

Several researchers who support the Extroversion-WM link have examined the role that dopamine might play in this relationship. Dopamine has particularly been implicated in attentional control, an important aspect of the executive control in Baddeley’s WM model. Arnsten (1998) proposes an inverted U curve to explain the relationship between dopamine and WM. If Extroversion is indeed associated with improved WM performance, it is possible that extroverts naturally have dopamine levels in the target range for maximum WM performance. This possibility was tested by Chavanon, Wacker, Leue, and Stemmler (2007). They divided participants into high and low Extroversion groups and then randomly assigned a placebo or dopamine receptor antagonist, sulpiride, which would decrease overall dopamine activity. In the placebo condition, extroverts showed increased cortical activity and performance in both high and low-load n-back tasks, but extroverts who had been given sulpiride performed worse on the WM task (Chavanon, Wacker, Leue, & Stemmler, 2007).

Caffeine, on the other hand, may facilitate dopamine release through its antagonist effect on adenosine (Smillie & Gokcen, 2010). Researchers have reported a positive effect of caffeine on WM performance (see Psychoactive Substances section of this paper), and this effect may be particularly strong for extroverts. Using a double-blind placebo controlled design, Smillie and Gokcen (2010) found that 200mg of caffeine improved n-back performance for extroverts, but not introverts, on a high load n-back task. This experiment was replicated with a relatively low dose of caffeine, 65mg in a cup of coffee, resulting in greater WM improvement for extroverts compared to introverts (Smith, 2013). Altogether, these studies support the hypothesis that dopamine plays an important role in WM performance and provide evidence for differences in WM processes between introverts and extroverts.

Conscientiousness and Neuroticism have been associated with WM performance as well. Conscientiousness is associated with persistence, reliability, and self-discipline and Neuroticism is associated with emotional instability, anxiety, and depression (Studer-Luethi et al., 2012). Overall, higher Neuroticism has been related to lower WM performance and higher Conscientiousness has been related to increased WM performance on an n-back task (Studer-Luethi et al., 2012).

Overall, researchers have reported intriguing relationships between certain personality traits, particularly Extroversion and WM. As shown in Table 1, we view personality as a relatively stable biological factor (i.e. chronic effect with low malleability). Additional research, both cognitive and physiological, will be required to fine-tune our understanding of the relationship between personality factors and WM.

### Mental Illness and Other Medical Conditions

A wide variety of mental illnesses have been associated with deficits in WM performance (see Table 2 for a list of conditions and applicable references). The deficits observed in people diagnosed with schizophrenia have received an enormous amount of attention in the literature. Not only are these impairments seen in people diagnosed with schizophrenia, but they have also been found in first degree relatives of individuals who have been diagnosed with a schizophrenia spectrum disorder. People with schizophrenia and people with schizotypal personality disorder have difficulty ignoring distracting stimuli and maintaining information in WM. These deficits may be attributed to dopamine function in the PFC (Barch et al., 2003), which then impairs functioning of the central executive in WM. Of note, not all studies have found a relationship between WM and risk for schizophrenia. Using a latent-variable approach, WM did not predict scores on a measure of schizotypy (Kane et al., 2016).

**Table 2:** Mental Diagnoses Associated With Impaired WM Performance

Disorder	References
Schizophrenia spectrum	Barch, 2003; Brahmbhatt, 2006; Callicott et al., 2003; Goldstein et al., 2011
Borderline personality	Hagenhoff et al., 2013; Lazzaretti et al., 2012; Stevens, Burkhardt, Hautzinger, Schwarz, & Unckel, 2004
PTSD	Galletly et al., 2008; Jelinek et al., 2008
OCD	Rampacher et al., 2010
Hoarding disorder	Ayers et al., 2013
Depressive disorders	Gohier et al., 2009; Pio de Almeida et al., 2012; Rose & Ebmeier, 2006
ADHD	Dowson et al., 2004
Autism	Cui, Gao, Chen, Zou, & Wang, 2010; Steele, Minshew, Luna, & Sweeney, 2007

In addition to disorders on the schizophrenic spectrum, several other mental disorders have been investigated for deficits in WM. Certain personality disorders have been associated with reduced WM (Coolidge, Segal, & Applequist, 2009), particularly borderline personality disorder. Anxiety disorders associated with reduced WM include post traumatic stress disorder (PTSD), obsessive compulsive disorder (OCD), and even hoarding disorder. Mood disorders that have been associated with WM deficits include major depression, postpartum depression, and social anhedonia. These disorders may influence WM through a mechanism of preoccupying thoughts, a possibility further discussed in the next section.

WM performance has been thoroughly examined in people who have been diagnosed with attention deficit hyperactivity disorder (ADHD). ADHD certainly impairs WM performance in children (Kasper, Alderson, & Hudec, 2012; Martinussen, Hayden, Hogg-Johnsen, & Tannock, 2005). However, there is some evidence that WM training can decrease ADHD-related symptoms (Klingberg et al., 2005; Klingberg, Forssberg, & Westerberg, 2002; Mezzacappa & Buckner, 2010). WM deficits are not always seen in autism, but can be observed in some high-load tasks.

In addition to mental illness, many other recognized disorders have been associated with impaired WM function (see Table 3 for a list of these conditions along with applicable references). Degenerative disorders, such as Alzheimer’s disease and Parkinson’s disease, decrease WM performance. In fact, levodopa (L-dopa) can improve WM performance in people who have been diagnosed with Parkinson’s (Lange et al., 1992; Malapalani, Pillon, Dubois, & Agid, 1994). WM is also impaired in people who have experienced traumatic brain injury (TBI).

**Table 3:** Medical Conditions Associated With Impaired WM Performance

Disorder	References
Alzheimer’s	Gagnon & Belleville, 2011; Huntley & Howard, 2010
Parkinson’s	Gilbert, Belleville, Bherer, & Chouinard, 2005; Owen, Iddon, Hodges, Summers, & Robbins, 1997
TBI	McDowell, Whyte, & D’Esposito, 1997
Fragile X	Baker et al., 2011; Munir, Cornish, & Wilding, 2000; Ornstein et al., 2008
Epilepsy	Longo, Kerr, & Smith, 2013
Multiple sclerosis	Parmenter, Shucard, Benedict, & Shucard, 2006
Chronic fatigue	Deluca et al., 2004
Spina bifida	Mammarella, Cornoldi, & Donadello, 2003

Fragile X syndrome, a neurodevelopmental disorder caused by too many codon repeats on the X chromosome, has been associated with both verbal and spatial WM deficits, regardless of task difficulty. WM is also impaired in children with epilepsy and spina bifida. Deficits in WM have been reported in people with multiple sclerosis and people diagnosed with chronic fatigue.

Many mental illnesses and general medical conditions have been associated with declines in WM, including genetic disorders and age-related disorders. Some of these have a clear biological basis, but others are not so definitive. Additional research is required to fully understand the mechanism of these deficits and develop methods to improve WM function in these groups. Even so, it would be prudent to screen for the aforementioned disorders in large-scale studies. As seen in Table 1, we rate mental illness as medium and medical condition as low in terms of malleability; the mental illnesses we list are treatable in many cases, while the medical conditions we list are generally more difficult to alleviate. Nevertheless, we see both mental and medical conditions as chronic in nature and due to largely biological factors, with a few exceptions related to preoccupying thoughts (e.g. OCD).

## Manipulated Factors

In this section, we will present a series of manipulated factors and their relationship to WM performance. These factors differ considerably from one another, but some similarities and patterns can be observed. For instance, the first seven factors that we describe are all associated with the concept of preoccupying thoughts. All the factors in the next section share one common feature; namely, their effect on WM performance can be manipulated.

### Emotion

Some factors have a fleeting effect on WM performance, as research on state-dependent influences (such as emotion and motivation) has shown. Emotion itself can impair cognition. For example, using an n-back task of faces, reaction time was slower for fearful faces compared to faces showing neutral expressions (Kensinger & Corkin, 2003). Daily emotional ups and downs can also influence WM performance. Brose, Lövdén, and Schmiedek (2014) found that people perform better on WM tasks on days they feel more positive affect compared to days they feel less positive affect. Negative affect is particularly associated with decreased initiative and WM performance (Brose, Schmiedek, Lövdén, & Lindenberger, 2012). As mentioned in the section on age-related declines in WM, elderly adults show enhanced WM performance when stimuli have positive emotional valance (Mikels, Larkin, Reuter-Lorenz, & Carstensen, 2005).

Researchers have also examined emotion and WM from a physiological perspective. Gray (2004) contends that emotion and cognitive processes are related and selectively influence each other. For instance, Gray (2001) induced approach or withdrawal type emotion and then tested participants on a verbal and spatial 2-back task. Withdrawal emotion selectively enhanced spatial WM while approach enhanced verbal WM performance. This makes theoretical sense because spatial ability and withdrawal/inhibition are both associated with right hemispheric function while verbal ability and approach behaviors are associated with left hemispheric function (Gray, 2001). Gray (2001) suggests that dopamine may modulate the effect of positive emotion on WM and that norepinephrine might modulate withdrawal on WM.

Motivation can also be considered a state-dependent influence on WM. Sanada, Ikeda, Kimura, and Hasegawa (2013) examined money as a motivator in a visual WM task and found that motivation improved visual WM in both high and low load tasks. Others have also concluded that motivation enhances WM (e.g., Gilbert & Fiez, 2004; Krawczyk, Gazzaley, & D’Esposito, 2007).

Emotion can clearly influence performance on WM tasks, with different emotions having different levels of impact. In general, negative emotion seems to have an impairing effect on WM; however, under certain circumstances, positive emotion and motivation can enhance WM performance. We view emotion as an acute effect, with a medium level of malleability, and attribute the cause of the effect on WM performance to the preoccupying thoughts that accompany strong emotion (Table 1). It is possible that preoccupying thoughts use up resources in the phonological loop, and this hypothesis will be discussed in the conclusion to this review.

### Stress and Anxiety

In contrast to purely state-level effects, anxiety can be viewed as either a state or trait-level influence on WM (Ilkowska & Engle, 2010). For instance, Sorg and Whitney (1992) examined the influence of both trait anxiety and stress on WM performance. Participants were divided into high/low anxiety groups and then exposed to either a stressful or non-stressful situation (a video game competition) before being tested on reading span. High trait anxiety people showed impaired verbal WM in the stress condition but not the low stress condition (Sorg & Whitney, 1992). The effect of trait anxiety is not limited to adults. Visu-Petra, Cheie, Benga, and Alloway (2011) tested preschoolers on both verbal and spatial WM tasks and found that high trait anxiety was associated with longer verbal WM response times and poorer accuracy. By contrast, state anxiety seems to impair spatial but not verbal WM, with high WM individuals impaired by induced anxiety the most (Shackman et al., 2006).

Rapee (1993) took a different approach and examined the effect of participation in a WM task on worry itself. Rapee (1993) asked participants to focus on an area of concern that they would be able to worry about for some time while either generating random letters, engaging in articulatory suppression, completing a visuospatial task, or generating random numbers on a key pad and then tested the change in worry. Tasks which involved the central executive and especially the phonological loop were able to reduce a worried state.

Multiple researchers have hypothesized as to the underlying mechanisms that allow anxiety to influence WM. Elliman, Green, Rogers, and Finch (1997) found that anxiety impairs processing ability in both the central executive and the phonological loop. They suggest that this impairment is due to pre-occupying anxious thoughts and subvocalization using up WM resources (Elliman et al., 1997). Others have concluded that anxiety has a greater effect on the phonological loop than the visuospatial sketchpad because worry-related thoughts consume cognitive resources (e.g. Derakshan & Eysenck, 2009; Eysenck, Derakshan, Santos, & Calvo, 2007; Rapee, 1993). Ashcraft and Kirk (2001) report that math anxiety impairs WM due to intrusive thoughts and worries about math itself. Evaluation anxiety has the same effect, and most likely operates on this same principle of consumed cognitive resources (Coy, O’Brien, Tabaczynski, Northern, & Carels, 2011).

Anxiety has a uniformly detrimental effect on WM performance, and the root of this effect is the use of attentional resources by preoccupying thoughts centered on the anxiety-producing stimulus. The effect of stress and anxiety on WM performance is generally acute, and there is a medium amount of malleability (Table 1).

### Dieting

Another interesting factor that has been associated with impaired WM performance is dieting; a deficit that seems particularly obvious in high load verbal tasks (Kemps, Tiggemann, & Marshall, 2005). Unexpectedly, this association does not seem to be due to weight loss or caloric restriction itself (Vreugdenburg, Bryan, & Kemps, 2003). Green and Rogers (1995) found that dieters performed worse on cognitive tasks, including attention, regardless of whether the dieter was successful or not in actually losing weight. Rather, the link between dieting and impaired WM seems to be due to preoccupying thoughts about food and body shape (Green et al., 2003; Kemps et al., 2005). These preoccupying thoughts reduce the amount of cognitive resources available for tasks that tap into WM (Kemps, Tiggemann, & Grigg, 2008; Kemps et al., 2005) because dieting requires self-monitoring and attention to maintaining dieting behaviors (Vreugdenburg et al., 2003).

Multiple studies have determined that dieting selectively interferes with the phonological loop but not the visuospatial sketchpad (Green et al., 2003), which makes sense considering the verbal nature of preoccupying thoughts. Vreugdenburg, Bryan, and Kemps (2003) compared dieters to non-dieters on tests of articulatory control and the phonological store. Dieters performed worse on overall central executive function and the phonological loop, particularly on the phonological store. A study by Shaw and Tiggemann (2004) compared past dieters, dieters, and non-dieters on phonological loop performance by examining the phonological similarity effect and articulatory control. Dieters did not show impaired performance on the phonological similarity effect, but were impaired on articulatory control, which was mediated by preoccupying thoughts (Shaw & Tiggemann, 2004).

The preoccupying thoughts associated with dieting are amplified in eating disorders, and cognitive performance suffers greatly (Mathias & Kent, 1998). In a study comparing two dozen women with anorexia nervosa to dieters and non-dieters, Kemps, Tiggemann, Wade, Ben-Tovim, and Breyer (2006) found that both people with anorexia nervosa and dieters performed worse on dual WM tasks due to preoccupying thoughts about food, weight, and body shape than non-dieters.

The effects of dieting may be both acute and chronic, with a high degree of malleability (Table 1). Similar to anxiety, the act of dieting seems to impair WM performance by consuming attentional resources through preoccupying thoughts.

### Craving Cigarettes

The negative nature of preoccupying thoughts extends past dieting. Smokers perform worse on WM task in which cues are smoking-related (Wilson, Sayette, Fiez, & Brough, 2007) and chocolate-cravers perform worse on WM tasks as well (Tiggemann, Kemps, & Parnell, 2010).

Smokers who abstain from smoking at least 12 hours, and are presumably craving that behavior, show impaired WM performance — a finding that can be reversed by allowing smokers to smoke or by giving them nicotine (Blake & Smith, 1997; Ernst, Heishman, Spurgeon, & London, 2001; Jacobsen et al., 2005). However, not all studies agree with these results (e.g., Park, Knopick, McGurck, & Meltzer, 2000).

Greenstein and Kassel (2009) suggest some of the inconsistency across studies is due to differences in methodology and the specific test that was used to assess WM. To address this possibility, they used verbal and spatial versions of the n-back and OSPAN. They found that smokers had significantly lower verbal WM than nonsmokers, both when they were permitted to smoke and when they were abstinent (Greenstein & Kassel, 2009), although there was no difference in verbal n-back performance between the smoking sessions. In addition, there was no difference in spatial WM between smokers who were craving smoking and those who were permitted to smoke. Craving smoking seemed to selectively impair verbal WM and not spatial WM, similar to the findings regarding dieting and the action of preoccupying thoughts in the phonological loop. Cravings are highly malleable and tend to be acute (Table 1), although additional research is required beyond cigarettes and chocolate (e.g. gambling).

### Stereotype Threat

Stereotype threat occurs when a person is made aware of a stereotype regarding a group of which they are a member – including groups based on gender, race, and income level – and feel that they are at risk for conforming to such stereotypes. Performance of those in negatively stereotype groups is hindered when the stereotype is present, causing anxiety (Steele, 1997). This stereotype threat can then impair performance on WM tasks. For example, Schmader and Johns (2003) induced a stereotype threat in women by highlighting gender differences in math ability and in Latinos by stressing ethnic group intelligence differences. OSPAN performance was significantly lower for both women and Latinos after receiving the stereotype threat compared to individuals who did not receive the negative stereotypes (Schmader & Johns, 2003). Stereotype threat is particularly detrimental in high load tasks. Beilock, Rydell, and McConnell (2007) tested women on high and low load tasks and found that women who were given a gender-based stereotype threat performed worse on high load problems compared to women who did not receive the threat.

Some researchers suggest that the negative effect of stereotype threat on WM performance can be increased by activating multiple stigmatized aspects of group membership (Forbes & Schmader, 2010). Tine and Gotlieb (2013) tested multiple stereotype threats based on gender, race, and income level and found that each of these stereotype threats decreased WM performance, but income-based effects were the strongest of the three. Individuals with all three stigmatized aspects of identity (i.e. a woman of a minority group in a low-income bracket) experienced greater deficits in WM after the threat compared to individuals who received just one or two types of threat.

Eliminating stereotype threat through counter-stereotype training is also possible, as demonstrated by Forbes and Schmader (2010). They retrained women to associate their gender with being good at math, which then increased performance on a complex span task. Specifically, women who were trained to associate women with being good at math had higher WM scores than women who were trained to associate men with being good at math (Forbes & Schmader, 2010).

Beilock, Rydell, and McConnell (2007) believe that stereotype threat specifically targets the phonological loop. However, others propose alternate explanations (e.g. the ‘mere effort’ hypothesis, Harkins, 2006), and some even question whether stereotype threats impair performance. Jamieson and Harkins (2007) report that women who received a stereotype threat regarding math and gender performed worse on some tasks and better on others, and they suggest that stereotype threats may motivate behavior by creating a situation in which a stereotyped individual wants to prove the stereotype wrong through their own efforts.

Despite this, the general consensus in this area seems to be that stereotype threats generally impair WM performance. A comparison of WM tasks that use verbal or spatial information is called for, and this type of experiment should be extended to multiple types of stereotype threat, as most of the studies have focused on gender thus far. Overall, stereotype threat produces a highly malleable, acute effect on WM performance (Table 1).

### Temperature

Schoofs, Wolf, and Smeets (2009) found that administering a cold pressor test (i.e. immersing one hand in ice water) impairs executive functioning in a WM task. Although Schoofs et al. tested the effects of acute stress, extremes in temperature might also affect WM performance. Sellaro, Hommel, Manai, and Colzato (2015) suggest that it may not be objective temperature that impairs WM so much as subjective temperature preferences. Using a 2 (temperature preference: cold vs. warm) x 2 (environment: cold vs. warm) design, they tested performance in different work environments. People who were tested in an environment contrary to their temperature preferences performed worse on a 2-back task compared to people were tested in their preferred temperature, regardless of whether it was warm or cold. Sellaro et al. (2015) suggest that subjective temperature preferences influence WM performance, but that objective temperatures may not.

Nevertheless, multiple studies have found a link between cold temperatures and impaired WM. These studies have specifically targeted tyrosine in their design. Tyrosine is a substrate for catecholamine synthesis in the brain, and is a precursor for the catecholamine neurotransmitters, dopamine and norepinephrine (O’Brien, Mahoney, Tharion, Sils, & Castellani, 2007). Because exposure to cold increases the activity of (and therefore depletes) catecholamines, several researchers have tested whether the administration of tyrosine can reverse the negative effect of cold-stress on WM. Mahoney, Castellani, Kramer, Young, and Lieberman (2007) found that post-cold pressor WM performance improved after the consumption of a food bar containing tyrosine. Mahoney et al. conclude that cold exposure degrades cognitive performance and tyrosine alleviates the cold-related WM deficits. Likewise, Shurtleff, Thomas, Schrot, Kowalski, and Harford (1994) found that administering tyrosine before immersion in cold water significantly improved accuracy on a WM task, possibly by preventing the cold stress-induced reduction in brain catecholamines.

The existing literature suggests cold temperatures negatively influence WM performance. The mechanism of this relationship might be stress, and is certainly related to levels of catecholamines in the brain. In Table 1, we describe temperature as an acute, highly malleable manipulated factor; however, the research in this area is sparse and too many questions remain for a firm conclusion as to the nature of this factor.

### Mindfulness

Although several of the factors discussed in the preceding section all seem to impair WM performance due to the presence of preoccupying thoughts, mindfulness meditation may improve WM performance by controlling and reducing preoccupying thoughts. Mindfulness meditation seeks to promote attentional control and awareness of internal and external experiences (Chambers, Lo, & Allen, 2008). In a mindful state, all thoughts are examined without judgement or reaction; they are simply allowed to exist. Mindfulness incorporates both the regulation of attention and an open and accepting attitude to mental activity (Chambers et al., 2008). Interestingly, the acceptance facet of mindfulness training has been more strongly linked to WM performance on an n-back task (Ruocco & Direkoglu, 2013), at least compared to present-moment awareness. This result seems surprising at first, but accepting one’s thoughts without judgement requires cognitive control, which may then be the link to WM performance.

Performance on WM tasks has been improved through both brief and intensive mindfulness training. Zeidan, Johnson, Diamond, David, and Goolkasian (2010) found that 20 min of mindfulness training for four days enhanced accuracy, but not speed, on an n-back task. However, they caution that this gain may not be a long term change in WM performance (Zeidan et al., 2010). Chambers et al. (2008) tested 20 novice meditators before and after participation in a 10-day intensive mindfulness meditation retreat. They found significant improvements in WM and sustained attention; specifically, those who received the training performed better on a task-switching task than a comparison group who did not complete the training.

Although these studies seem like a promising avenue to improve WM, they certainly require replication. Researchers should also address possible demand characteristics in future studies by including placebo control groups. In addition, many unanswered questions remain. For instance, how long does training influence WM? Does the intensity and duration of mindfulness meditation training influence the strength and duration of the effect? These are important questions that should be addressed in the future. Because few studies have been conducted in this area and because of uncertainty regarding the duration of effect (Table1), we cannot make firm conclusions about the effect of mindfulness on WM at this time.

### Practice

Perhaps the most intriguing factor that can alter WM performance, from the perspective of both basic and applied researchers, is regular practice on WM tasks. Multiple researchers have reported improvements in WM performance after training on a WM task (Bomyea & Amir, 2011; Dahlin, Nyberg, Bäckman, & Neely, 2008; Klingberg et al., 2005; Li et al., 2008; Redick et al., 2012; Sprenger et al., 2013; Zinke et al., 2014), and this result has created great interest in the effect of practice on WM performance.

However, one major controversy in the literature surrounds the claim that WM training can ‘transfer,’ or create gains in other cognitive functions. Much of this debate was stirred by a report that 8-19 days of training on an adaptive dual n-back resulted in gains on a test of fluid *g* (Jaeggi, Buschkuehl, Jonides, & Perrig, 2008). Since then, little support for this claim has been found, and a multitude of studies have failed to replicate any effect of WM training on *g*F or general cognitive ability (Brehmer, Westerberg, & Bäckman, 2012; Chooi & Thompson, 2012; Holmes, Gathercole, & Dunning, 2009; Redick et al., 2012; Richmond, Morrison, Chein, & Olson, 2011; Sprenger et al., 2013; Zinke, Zeintl, Eschen, Herzog, & Kliegel, 2012). In critical reviews of the literature, both Shipstead, Redick, and Engle (2012) and Melby-Lervåg and Hulme (2013) conclude that WM training does not increase intelligence.

Beyond fluid intelligence, though, some researchers claim that WM training can transfer to other tests of WM, such as complex span tests (Holmes, Gathercole, & Dunning, 2009; Richmond, Morrison, Chein, & Olson, 2011; Schmiedek, Lövdén, & Lindenberger, 2010). Even this is a questionable finding, as multiple studies (Buschkuehl et al., 2008; Jaeggi, Buschkuehl, et al., 2010; Jolles, Grol, Van Buchem, Rombouts, & Crone, 2010; Li et al., 2008; Redick et al., 2012; Sprenger et al., 2013) have not replicated this result.

In addition to transfer, the duration of training gains has caused considerable discussion. Gains in WM performance after training have remained in six (Jolles et al., 2010) to nine month (Zinke et al., 2014) follow ups. However, Buschkuehl et al. (2008) found that increases in visual WM gained from training disappeared at a one year follow up. In addition, transfer gains may not hold up over time (Borella, Carretti, Riboldi, & De Beni, 2010).

Research on the effects of WM training has been conducted on a range of age groups, from children (Klingberg et al., 2002, 2005) and young adults (Dahlin et al., 2008; Jaeggi et al., 2008) to adults and the elderly (Borella et al., 2010; Dahlin et al., 2008). In addition, some researchers report gains in WM performance in children with ADHD (Klingberg et al., 2005) and in adult neuropsychological patients following strokes (Westerberg & Klingberg, 2007), indicating that some populations gain more from WM practice than others and that WM practice may be a viable tool to improve cognitive function in people with WM impairment. Nevertheless, there may be pervasive methodological concerns with studies of WM training (Shipstead, Redick, & Engle, 2012) and thus WM training remains a controversial topic in the field at this time. Table 1 reflects this controversy, and both the length of effect and underlying mechanism are listed as uncertain.

### Sleep

Sleep deprivation has a consistently negative effect on WM performance (Lieberman, Tharion, Shukitt-Hale, Speckman, & Tulley, 2002). Casement, Broussard, Mullington, and Press (2006) found that people who were chronically sleep deprived (i.e. regularly experiencing less than half of normal sleep times) performed worse on a task that required WM than non-deprived participants. This deficit is particularly strong under greater WM loads; Cellini, de Zambotti, Covassin, Sarlo, and Stegagno (2014) report that people with chronic insomnia performed just as well as healthy controls on an easier WM task but performed significantly worse on a high load WM task.

The negative effect of sleep deprivation on WM has also been shown in adolescent populations. Gradisar, Terrill, Johnston, and Douglas (2008) asked teenagers aged 13-18 to complete the OSPAN and self-report their sleep time and found medium to large effect sizes of sleep on WM scores. Even younger children perform worse on WM tasks when sleep deprived; significant WM deficits have been found in sleep-deprived 6-14 year olds (Steenari et al., 2003).

In contrast to studies examining sleep deprivation, getting a good night’s sleep seems to have a positive effect on the relationship between WM and memory. A medium positive correlation between performance on the OSPAN and a general memory task was found after participants had slept but this result was not found in those who had remained awake (Fenn & Hambrick, 2012).

Several researchers have approached the sleep-WM relationship from a physiological perspective. Choo, Lee, Venkatraman, Sheu, and Chee (2005) used an n-back task to test WM in participants who had been sleep deprived for 24 hours. The participants showed decreases in both response time and accuracy compared to participants who were not sleep deprived. Some load dependent differences were seen in the left PFC, right parietal, anterior medial PFC, and left anterior cingulate cortex (Choo et al., 2005). The left anterior cingulate cortex also showed increased activity due to sleep deprivation, but the right frontal gyrus and right insula were active in sleep-deprived participants only in the higher load conditions (Choo et al., 2005). In a follow-up study, Chee et al. (2006) found that sleep deprivation resulted in reduced activity in fronto-parietal regions. Interestingly, sleep-deprived individuals who showed greater left fronto-parietal activity showed superior WM performance over people without this pattern of brain activity (Chee et al., 2006).

As obvious as the relationship between sleep deprivation and WM may seem, and considering the frequent use of college students in psychological research (a group historically known for sleep deprivation), it is surprising that this factor is not taken into consideration more often by researchers. Sleep deprivation can become an influential extraneous variable in such studies and it would be prudent to assess sleep deprivation in WM studies in general. We view sleep as having an acute and highly malleable effect on WM performance, although the exact mechanism of the effect is uncertain (Table 1).

### Bilingualism

Although some people may view bilingualism as an individual difference due to stability over time, we have included it in this section because it at least possesses the potential for manipulation (i.e. an individual may choose to learn a second language whereas they cannot choose their age or level of intelligence). Much of the research on bilingualism and WM has been conducted by linguists, who have found some intriguing results. Two interesting findings are the strong correlation between first language WM and second language WM, and the strong correlation between baseline WM performance and foreign language proficiency (Alptekin & Ercetin, 2010, 2011; Daneman & Carpenter, 1980; Harrington & Sawyer, 1992; Miyake & Friedman, 1998; Osaka, Osaka, & Groner, 1993; Walter, 2004). These results have led several researchers to claim that WM plays an important role in the acquisition of a second language (Mackey, Philp, Egi, Fujii, & Tatsumi, 2002; Williams, 1999) and in determining second language aptitude (Ellis, 2001; Miyake & Friedman, 1998; Sawyer & Ranta, 2001).

Some investigators have looked at WM and bilingualism across the lifespan. Several studies have found no difference in WM performance between monolingual and bilingual children (Bialystok & Feng, 2009; Bonifacci, Giombini, Bellocchi, & Contento, 2011). Although Kudo and Swanson (2014) found no evidence that bilingual children have an advantage in WM, they did find that children transitioning from being monolingual to bilingual or from being dominant in one language to more equal in their bilingualism show enhanced WM, particular in the executive component. However, studies that report no differences in WM performance between monolingual and bilingual children may have used low-load tasks, and the difference between these groups may be more evident at higher cognitive loads. For instance, bilingual children did outperform monolingual children on a WM task in one study, although this advantage was more prominent in the more demanding, higher load tasks (Morales, Calvo, & Bialystok, 2013). Differences between spatial and verbal WM have also been investigated; bilingual participants outperform monolingual participants in spatial WM, but not verbal WM, across the adult life span (Luo, Craik, Moreno, & Bialystok, 2013).

The phonological loop may be a significant factor in second language learning outcomes (Baddeley, 2003; Service, 1992). As the phonological loop deals with verbal information, it makes sense that it would be implicated in language learning. However, other researchers suggest that WM performance in a second language is due to the executive component of WM, and is independent of the phonological loop (Lee Swanson, Orosco, & Lussier, 2015). This debate requires further investigation.

Regardless of the role individual WM components play, it seems clear that WM is closely related to learning a second language. Sawyer and Ranta (2001) view WM as a system responsible for integrating the different components of foreign language. For instance, navigating between two languages requires holding some linguistic information in mind while manipulating another language, which certainly mirrors the manipulate/maintain definition of WM (Bialystok, 2011). Nevertheless, many unanswered questions remain. One question centers on the issue that some researchers treat WM as a cause of second language success and others treat it as an effect. More specifically, some researchers seem to study the role baseline WM has in increasing success in second language acquisition, whereas others examine differences in WM between monolingual and bilingual groups. Whether high WM improves the chances of becoming bilingual or learning a foreign language improves WM performance is debatable and requires additional research. Certainly the effect is chronic, and we assigned a medium degree of malleability due to the effort needed to become bilingual (Table 1).

### Musical Training

Musical training has also been investigated for its beneficial effect on WM task performance (Besson, Schön, Moreno, Santos, & Magne, 2007; Williamon & Egner, 2004). Long-term music training has been associated with greater auditory and spatial WM; specifically, years of music training and WM performance are positively correlated (George & Coch, 2011).

Both children and adults who have received music training outperform non-musicians on various measures of WM, both verbal and spatial (Franklin et al., 2008; Fujioka, Ross, Kakigi, Pantev, & Trainor, 2006; Parbery-Clark, Skoe, Lam, & Kraus, 2009). Lee, Lu, and Ko (2007) tested 40 middle-school age children and 40 college age adults on the effects of musical training on WM using span tasks. Half of the participants in each group had received several years of musical training in the past and could play at least one musical instrument. Participants were matched on both intelligence and level of education. The musically trained children and adults performed better than the untrained participants on tasks designed to measure phonological storage (Lee et al., 2007). The musically trained children performed better than the untrained children on measures of executive control and visuospatial storage. However there was no difference between the two adult groups, suggesting that the effects of musical training on WM may depend both on age and the degree of music training received (Lee et al., 2007).

Unfortunately, the studies conducted thus far used convenience samples of people who have already received musical training. Similar to bilingualism, this is a potential problem of self-selection: individuals with higher WM may be naturally drawn to music or are more persistent in practicing an instrument. A longitudinal study, with a true experimental design, is needed to address this issue. As with bilingualism, the effect of musical training on WM performance is chronic and somewhat malleable due to the effort required for musical success (Table 1).

### Altitude/Hypoxia

One of the more unusual factors in this review is altitude (i.e. how far above sea level one is). Yan, Zhang, and Gong (2011) found that people who lived at high altitudes performed worse than low altitude residents on a WM task. This difference held for both response times and accuracy. In another study, they compared sea level participants to natives of Tibet and found that the high altitude Tibetans performed worse on both verbal and spatial WM tasks, again showing deficits in response time (Yan, Zhang, Shi, Gong, & Weng, 2010). Even brief exposure to high altitudes has shown a suppressing effect on WM performance (see Virués-Ortega, Buela-Casal, Garrido, Alcázar, 2004, for a review).

A possible cause of this phenomenon is hypoxia— the level of oxygen in the air people breathe. Oxygen is less abundant at higher elevations. A few studies have examined the negative effect of hypoxia during sleep on WM (e.g., Biggs et al., 2011; Weiss et al., 2009) and during exercise (Komiyama et al., 2015), but these studies introduce other variables to consider. As seen in Table 1, we rate this factor as highly malleable, biological in cause, and having both an acute and chronic effect. However, too few studies have been conducted to make any firm conclusions about the nature of the effect. Though empirical work is sparse in this area, the existing studies open the door to explore other environmental factors, such as barometric pressure or humidity.

### Exercise

Some controversy surrounds the effect of exercise on WM performance. Some researchers have reported that exercise has a negative effect on WM, others find no effect at all, whereas several others report a WM advantage from engaging in exercise. We will review research from each of these perspectives.

First, several groups of researchers report no effect, or even a negative effect, of exercise on WM. For example, Dietrich and Sparling (2004) found that WM was impaired during prolonged moderate exercise, whereas Lambourne, Audiffren, and Tomporowski (2010) found no change in WM between rest and cycling (exercise) conditions. The effect of exercise on children’s WM is also debated. Drollette, Shishido, Pontifex, and Hillman (2012) found no change in WM during moderate walking in 9-11 year olds, yet Soga, Shishido, and Nagatomi (2015) report that moderate walking results in slower response times on a spatial n-back task in 15-16 year olds.

Other researchers reported that exercise improves response times and accuracy on WM tasks. Hogan, Mata, and Carstensen (2013) found that moderate cycling resulted in faster reaction time on a 2-back WM task for people ages 19-93. WM has also been assessed during exercise (as opposed to post-exercise). Martins, Kavussanu, Willoughby, and Ring (2013) report that moderate cycling increased accuracy on concurrent high load WM tasks.

These varying results are difficult to explain. Soga, Shishido, and Nagatomi (2015) suggest that the effects of exercise are short-lived, so that a delay in testing might miss an effect. Baseline fitness is also an issue, as people in better shape may have better attentional control, which is an important factor in WM. This hypothesis was supported by a study conducted by Clarkson-Smith and Hartley (1989), in which high-exercise adults outperformed sedentary adults on three span tasks, even when controlling for verbal intelligence. However, even long term effects of exercise on WM are uncertain; a more recent study found that a four-week regimen of aerobic exercise, strength training, and stretching resulted in no difference in WM performance. (Nouchi et al., 2014). Given the wide variety of results, additional research on exercise and WM is needed and both the timing of the WM test and baseline fitness should considered.

### Diet

In addition to the act of dieting (previously discussed in *Dieting* section), diet itself can have a measurable influence on WM performance. The effects of sugar (glucose), dairy, protein, and fat have been examined, with the bulk of the research conducted on the effect of glucose.

Glucose consumption results in a relatively fast, short term improvement in WM (Scholey, Harper, & Kennedy, 2001). This effect is so rapid that researchers who have not found an effect of glucose on WM have concluded that delay in testing was to blame (Sünram-Lea, Foster, Durlach, & Perez, 2002). Importantly, the positive effect of glucose on verbal and spatial WM is the result of both dosage and recency of administration, and this effect is most pronounced in higher load conditions (Owen, Scholey, Finnegan, Hu, & Sunram-Lea, 2012). Conversely, induced acute hypoglycemia can cause impaired WM performance on span tests (Sommerfield, Deary, McAulay, & Frier, 2003).

These results are particularly interesting in light of studies that characterize both self-control and attentional control as finite energy reserves that rely on glucose (Gailliot et al., 2007). Some researchers maintain that the effect of glucose is age dependent; that is, younger people need less glucose to see an effect on WM performance (Messier, 2004).

Glucose paired with other substances can have a measureable effect WM as well. Using a double-blind design, Adan and Serra-Grabulosa (2010) found that participants who received both glucose and caffeine after a fasting period showed better attentional control and verbal memory compared to people who received only glucose or caffeine alone. Combining glucose and caffeine has also been found to improve performance on both verbal and spatial n-back tasks of various loads (Giles et al., 2012).

Besides glucose, dairy intake (which contains lactose) has also been associated with improvements in WM performance. Crichton, Murphy, Howe, Buckley, and Bryan (2012) conducted a within-subjects study of obese adults with habitually low dairy intake (i.e. less than 2 servings a day). Participants consumed a high dairy diet for six months and a low dairy diet for an additional six months. Spatial WM was slightly better after six months of high dairy (Crichton et al., 2012). Crichton et al. (2012) propose that low-fat dairy’s benefit to cardiovascular functioning might be boosting cognitive function, although high intake of full-fat dairy is associated with cognitive decline.

The effect of glucose has also been compared to protein and fat. Jones, Sunram-Lea, and Wesnes (2012) tested spatial and verbal WM at baseline, 15 min, and one hour after consuming either 40g glucose, 40g protein, 16g fat, or a placebo drink. Attention was better 15 min after consuming fat or glucose drinks, WM improved 15 min after protein consumption, and WM decreased 60 min after glucose (Jones et al., 2012). The authors conclude that different macronutrients have time-dependent effects on WM (Jones et al., 2012). This conclusion was supported by a study that compared a slow-release sucrose with lactose and glucose. Participants who consumed milk with sucrose performed best on a WM task (Taib, Shariff, Wesnes, Abu Saad, & Sariman, 2012).

Finally, a carbohydrate-rich breakfast has been shown to increase WM performance. Smith, Clark, and Gallagher (1999) compared WM performance on participants who consumed cereal for breakfast versus participants who did not eat breakfast. Consuming cereal for breakfast resulted in increased performance on a spatial WM task (Smith et al., 1999).

Many of the effects of diet seem to be short term. Glucose seems to confer short term benefits to WM, including when administered with caffeine. Protein, fat, and carbohydrates have also been linked to WM performance, but these studies require replication. In addition, more long-term studies are needed to determine if general diet has a long term effect on WM. Though we label diet as biological in cause and highly malleable (Table 1), the existing literature is too small to make firm conclusions about the effect of diet on WM performance at this time.

### Psychoactive Substances

A wide variety of drugs (i.e. external substances that are capable of crossing the blood brain barrier and influencing cognitive function) have been tested for their effect on WM (see Barch, 2004, for a review). Because of the amount of literature in this field, it is beyond the scope of this paper to provide a thorough review. Instead, representative articles have been chosen for several main psychoactive substances and additional resources are provided in the supplement to this article.

Both agonists and antagonists have been examined for their influence on WM. For instance, visuospatial WM is influenced by noradrenaline agonists in that both too high and too low doses cause impairment, forming an inverted U (Arnsten & Robbins, 2002). Dopamine agonists (such as amphetamine, methylphenidate, pergolide, and low doses of bromocriptine) improve spatial WM, although these effects seem to be susceptible to baseline differences (Barch, 2004; Luciana & Collins, 1997; Muller, von Cramon, & Pollmann, 1998). Cholinesterase inhibitors improve WM (Barch, 2004). There is minimal evidence that noradrenergic agonists and NMDA receptor agonists improve WM in humans, despite the strong effect seen in rats for both of these drug types (Barch, 2004). NMDA receptor antagonists, on the other hand, have been shown to impair WM (Adler, Goldberg, Malhotra, Pickar, & Breier, 1998).

One drug that undeniably has an influence on human cognition is caffeine, also known as methyltheobromine (Amendola, Gabrieli, & Lieberman, 1998; Fredholm, Bättig, Holmén, Nehlig, & Zvartau, 1999). Caffeine binds to two types of adenosine receptors in the brain and blocks them in an antagonistic way, which then increases alertness and concentration (Fredholm et al., 1999). The ability to concentrate - to control attention - is a key facet of WM, so it is perhaps no surprise that caffeine should have a positive effect on WM performance.

Indeed, nearly every study conducted on caffeine has found increased attention and WM as a result of consumption, with few exceptions (e.g. Smith, Clark, & Gallagher, 1999). Even habitual caffeine consumers still show increased WM performance after caffeine consumption (Addicott & Laurienti, 2009). In a study of male moderate caffeine users, Koppelstaetter et al. (2008) found that men in the caffeine condition showed increased activity in the medial PFC – often associated with attention, executive functions, maintenance in WM, planning, and updating - during a verbal 2-back task. In a study comparing a placebo to 100mg, 200mg, and 300mg of caffeine in sleep-deprived Navy SEALs, Lieberman, Tharion, Shukitt-Hale, Speckman, and Tulley (2002) conclude that 200mg of caffeine is optimal for performance on a spatial WM task. Past this amount, the effects do not significantly improve with dosage.

Several substances have been found to temporarily suppress WM (see Table 4 for a list of substances and references). Several of the effects appear to be load dependent, with high load task showing the greatest levels of WM impairment (i.e. alcohol, cannabis, MDMA). Not all psychoactive substances may influence WM; Colzato, Huizinga, and Hommel (2009) compared recreational cocaine users who had been abstinent for one week to a matched group of non-users and found no impairment in either the maintenance or monitoring of information in WM.

**Table 4:** Psychoactive Substances Associated With Impaired WM Performance

Substance	References
Diphenhydramine (Antihistamine)	Gevins, Smith, & McEvoy, 2002
Alcohol	Gevins et al., 2012
Cannabis	Ilan, Smith, & Gevins, 2004
MDMA (Ecstasy)	Fisk, Montgomery, & Hadjiefthyvoulou, 2011; Wareing, Fisk, Murphy, & Montgomery, 2004
Psilocybin	Wittmann et al., 2007

One study has examined the effect of Modafinil, a stimulant (but not an amphetamine) used in the treatment of narcolepsy and ADHD, using a double blind placebo-controlled design. Modafinil did not affect attentional control, but improved accuracy in the high load condition of a spatial WM task (Müller, Steffenhagen, Regenthal, & Bublak, 2004). It is of note that lower performing participants saw the greatest effect of drug (Müller et al., 2004). Considering the WM deficits associated with ADHD (see Mental Illness and Other Medical Conditions section), it is perhaps no surprise that medications designed to treat this disorder would improve WM performance.

Finally, emerging evidence suggests that certain herbal compounds can influence WM performance. In a study of thirty-two healthy adults, consumption of American ginseng improved WM performance on the Corsi block test at one, three, and six hours post administration, but performance on an n-back task was not affected (Scholey et al., 2010). Even a single dose of American ginseng has been shown to improve WM performance (Reay, Scholey, & Kennedy, 2010). Wesnes, Ward, McGinty, and Petrini, (2000) found improved WM in a 14-week double blind study of 256 people taking both 60mg ginko biloba and 100mg ginseng. Ginko and ginseng improved both spatial and verbal WM compared to baseline and the placebo group, although spatial WM saw greater increases, within hours of administration and over the fourteen weeks the study was conducted (Wesnes et al., 2000).

As previously stated, the sheer volume of research that has been conducted on psychoactive substances precludes a thorough review in this paper. Nevertheless, it is apparent that a wide variety of substances can influence performance on WM tasks. Currently, stimulants seem to have the greatest positive effect on WM while most other recreational substances seem to have a negative effect, although task load certainly plays a role. Psychoactive substances can have both acute and chronic effects and are highly malleable (Table 1), but a great deal of variation exists between the effects of individual drugs.

### Brain Stimulation

Several dozen studies have investigated the effect of brain stimulation on WM. The two main forms of stimulation that have been investigated are transcranial direct current stimulation (tDCS) and repeated transcranial magnetic stimulation (rTMS). tDCS is a procedure in which a weak electrical current is applied to the brain through two electrodes, an anode and a cathode. The goal of this procedure is to selectively increase or decrease cortical activity (Nitsche & Paulus, 2000; Nitsche et al., 2003). The electrical field generated by tDCS is direct but low-intensity (Datta et al., 2009). rTMS, on the other hand, uses a magnetic coil to create a strong magnetic field. When the coil is applied to the scalp, it disrupts brain activity in the cortex by depolarizing the neurons in that area. This technique may either facilitate or inhibit brain activity, depending on the frequency of stimulation (Brunoni & Vanderhasselt, 2014).

Both tDCS and rTMS are non-invasive procedures, and their effects on cortical activity are temporary. For this reason, they have been used extensively in research, including studies that stimulate the dlPFC to influence WM performance. The dlPFC is involved in both updating goal representations and other task-related demands (Barch, Sheline, Csernansky, & Snyder, 2003; D’Esposito, Postle, & Rypma, 2000; D’Esposito et al., 1995).

Nearly all of these types of studies use the n-back as their measure of WM. Unfortunately, most studies suffer from small sample size. For this reason, Brunoni and Vanderhasselt (2014) conducted a meta-analysis of twelve different studies that described 33 experiments to determine the influence of tDCS and rTMS on WM. Based on this meta-analysis, they conclude that rTMS of the dlPFC significantly improves WM performance. Although tDCS significantly improved reaction time, it did not influence accuracy (Brunoni & Vanderhasselt, 2014).

This result indicates that brain stimulation has a direct and measureable influence on WM performance. Though rTMS is already used in some clinical settings (e.g. as a treatment for major depressive disorder), there is some interest in using tDCS and rTMS in clinical populations that suffer from impaired WM, such as schizophrenia. Brain stimulation is a promising area for future study, and it would greatly benefit from additional studies using larger and more varied populations.

## Conclusions and Future Directions

This paper was written with several aims: to provide a review of past research, to serve as a resource for new researchers, to identify areas requiring additional research, and to provide a framework to classify these factors. As a result of these goals, we pulled many disparate areas of research together and highlighted some important considerations for researchers who frequently use WM tasks in their studies. As a reminder, there is a supplementary list of articles, arranged by topic, for each of the factors reviewed in this paper.

One of our primary goals was to provide a framework for classifying factors that influence WM. We divided factors along the lines of subject and manipulated factors, which seemed a logical first step in considering relationships between factors. Nevertheless, other possible frameworks may considered: duration of the effect, the malleability of the factor, the presumed mechanism that causes the variance in WM, and the level of consistency in the literature (see Table 1).

First, the duration of the effect on WM may be viewed as either acute or chronic. Chronic factors, such as intelligence and personality, are relatively stable over time. This causes their effect on WM to also be stable over time. Acute factors, such as emotional state and brain stimulation, are temporary, as is their effect on WM. Several factors are uncertain in terms of duration of the effect. These factors include dieting, mindfulness, practice, altitude, exercise, diet, and drug use. Each of these factors would greatly benefit from carefully designed, longitudinal studies.

Malleability refers to the likelihood of a factor changing, or being altered by external circumstances. Table 1 assigns low, medium, and high values of malleability to each factor, although these are best guesses based on knowledge of the field. Certain factors are nearly impossible to alter while other factors are easily changeable. Most of the highly malleable factors allow for personal choice, either by the individual or an experimenter. For example, a person can choose to exercise just as easily as an experimenter can assign a person to an exercise condition within a study. Of greatest importance, however, several factors that have detrimental effects on WM are malleable (e.g. stress, sleep, stereotype threat, and certain mental illnesses). This gives hope that interventions or therapies designed to improve or ameliorate the effects of these factors will have the side effect of improving WM performance.

A third possible framework relies on the underlying mechanism of each factor. Several factors are presumably biological in origin, such as intelligence, age, diet, brain stimulation, and use of psychoactive substances. Even personality has been investigated for its biological underpinnings, and the role of dopamine has certainly been implicated. Several studies have highlighted areas of the brain, such as the dlPFC, as important to WM, and have examined overlap with other factors, such as critically listening to music. Nevertheless, there seems to be a disconnect between neurological and behavioral studies of WM. As an example, the majority of neurological studies of WM use the n-back task. Though the n-back and complex span tasks both presumably measure WM, they are weakly correlated with each other and may actually measure slightly different aspects of WM itself (Kane, Conway, Miura, & Colflesh, 2007). Although a great deal of time and energy has been spent on investigating the underlying mechanisms of many of the factors reviewed in this paper, the cause of effect for several factors is unknown. These factors include mental illness, stereotype threat, temperature, practice, sleep, bilingualism, musical training, and exercise. Each of these factors is somewhat uncertain in their mechanism(s) and additional study is needed.

An additional framework for organizing the factors reviewed in this study is the level of consistency in the literature. Although several factors have been thoroughly investigated, questions about the nature of their effect remain. These factors include gender, personality, craving, practice, and exercise. Additional research may improve consensus in the field, and these factors would also benefit from thorough meta-analyses. In addition, some factors simply need more research in general, as too few studies have been conducted to make an assessment about consistency. These factors include temperature, mindfulness, musical training, altitude, and diet.

One important thread that connects multiple sections of this paper, and deserves special mention, is preoccupying thoughts. Intrusive thoughts are implicated in several types of mental illnesses, stress, anxiety, dieting, craving, and possibly stereotype threat. Preoccupying thoughts may selectively impair WM by using up resources in the phonological loop. Training to control preoccupying thoughts— for example, with mindfulness meditation— may diminish their effect, but additional research is needed to determine how effective this training is in the long term and if the training is truly effective at improving WM performance. An alternate to mindfulness meditation might be training on thought suppression. By increasing an individual’s ability to suppress intrusive thoughts, the harmful effects of those thoughts on WM might be reduced or eliminated. Future investigations might examine the role of preoccupying thoughts in terms of theoretical models of WM. For instance, using the Baddeley and Hitch (1974) model, preoccupying thoughts might either be a function of misallocation of attentional resources by the central executive or an impairment of the phonological loop.

Another general trend to note is the difference between high and low load tasks. In addition to differences between using verbal and spatial stimuli, researchers can also choose task difficulty by manipulating the task parameters. Increasing cognitive load may have a differential impact on the measurement of WM, as several factors (e.g., dieting, stereotype threat, sleep, and some psychoactive substances) seem to impair WM only, or especially, in high load tasks. Load-dependent influences for each of the factors in this study should be assessed in the future.

Finally, we have used this review to demonstrate the many possible sources of variance in WM performance. It behooves researchers to consider the sheer breadth and scope of variables that can influence WM, and to take more of a variance accounting approach in the measurement of this important construct. Several factors are quite easy to assess, as a researcher may easily include basic questions about them in a brief initial survey or include a short personality survey. Furthermore, some factors are more important to assess for certain populations, such as sleepiness in college students. In fact, the time of day and sleepiness in college students may explain some of the contradictory results between several of the studies described in this paper.

Understanding and accounting for the many factors that can influence WM performance will improve the quality and validity of our measurements. Of most importance, researchers who study WM or use it their research should understand that performance on WM tasks is not necessarily as stable as one would like to think, and multiple measures and measurements should be collected when possible. As Schmader and Johns (2003, p. 450) note in their study of stereotype threat, manipulations that “can produce situational reductions in WM underscores the need for researchers to use caution when assuming that such measures index a stable and unchanging ability.”

## References

[r1] Ackerman P. L. Beier M. E. Boyle M. O. (2005). Working memory and intelligence: The same or different constructs? Psychological Bulletin, 131, 30–60. doi:. 10.1037/0033-2909.131.1.30 15631550

[r2] Adan A. Serra-Grabulosa J. M. (2010). Effects of caffeine and glucose, alone and combined, on cognitive performance. Human Psychopharmacology, 25, 310–317. doi:. 10.1002/hup.1115 20521321

[r3] Addicott M. A. Laurienti P. J. (2009). A comparison of the effects of caffeine following abstinence and normal caffeine use. Psychopharmacology, 207(3), 423–431. doi:. 10.1007/s00213-009-1668-3 19777214 PMC2941158

[r4] Adler C. M. Goldberg T. E. Malhotra A. K. Pickar D. Breier A. (1998). Effects of ketamine on thought disorder, working memory, and semantic memory in healthy volunteers. Biological Psychiatry, 43, 811–816. doi:. 10.1016/S0006-3223(97)00556-8 9611670

[r5] Alloway T. P. (2010). Working memory and executive function profiles of individuals with borderline intellectual functioning. Journal of Intellectual Disability Research, 54, 448–456. doi:. 10.1111/j.1365-2788.2010.01281.x 20537050

[r6] Alptekin C. Ercetin G. (2010). The role of L1 and L2 working memory in literal and inferential comprehension in L2 reading. Journal of Research in Reading, 33, 206–219. doi:. 10.1111/j.1467-9817.2009.01412.x

[r7] Alptekin C. Ercetin G. (2011). The effects of working memory capacity and content familiarity on literal and inferential comprehension in L2 reading. TESOL Quarterly, 45, 235–266. 10.5054/tq.2011.247705

[r8] Amendola C. A. Gabrieli J. D. E. Lieberman H. R. (1998). Caffeine’s effects on performance and mood are independent of age and gender. Nutritional Neuroscience, 1, 269–280. 10.1080/1028415X.1998.11747237 27414696

[r9] Arnsten, A. F., & Robbins, T. W. (2002). Neurochemical modulation of prefrontal cortical function in humans and animals. In D. T. Stuss & R. T. Knight (Eds.), *Principles of frontal lobe function* (pp. 51-84). 10.1093/acprof:oso/9780195134971.003.0004

[r10] Arnsten A. F. T. (1998). Catecholamine modulation of prefrontal cortical cognitive function. Trends in Cognitive Sciences, 2, 436–447. doi:. 10.1016/S1364-6613(98)01240-6 21227275

[r11] Ashcraft M. H. Kirk E. P. (2001). The relationships among working memory, math anxiety, and performance. Journal of Experimental Psychology: General, 130, 224–237. doi:. 10.1037/0096-3445.130.2.224 11409101

[r12] Ayers C. R. Wetherell J. L. Schiehser D. Almklov E. Golshan S. Saxena S. (2013). Executive functioning in older adults with hoarding disorder. International Journal of Geriatric Psychiatry, 28, 1175–1181. doi:. 10.1002/gps.3940 23440720 PMC4037916

[r13] Babcock R. L. Salthouse T. A. (1990). Effects of increased processing demands on age differences in working memory. Psychology and Aging, 5, 421–428. doi:. 10.1037/0882-7974.5.3.421 2242246

[r14] Baddeley, A. D. (1986). *Working memory.* New York, NY, USA: Oxford University Press.

[r15] Baddeley A. D. (2000). The episodic buffer: A new component of working memory? Trends in Cognitive Sciences, 4, 417–423. 10.1016/S1364-6613(00)01538-2 11058819

[r16] Baddeley A. D. (2003). Working memory and language: An overview. Journal of Communication Disorders, 36, 189–208. doi:. 10.1016/S0021-9924(03)00019-4 12742667

[r17] Baddeley A. D. Hitch G. (1974). Working memory. Psychology of Learning and Motivation, 8, 47–89. 10.1016/S0079-7421(08)60452-19635207

[r18] Baddeley, A. D., & Logie, R. H. (1999). Working memory: The multiple-component model. In A. Miyake & P. Shah (Eds.), *Models of working memory: Mechanisms of active maintenance and executive control* (pp. 28-61). Cambridge, United Kingdom: Cambridge University Press.

[r19] Baker S. Hooper S. Skinner M. Hatton D. Schaaf J. Ornstein P. Bailey D. (2011). Working memory subsystems and task complexity in young boys with Fragile X syndrome. Journal of Intellectual Disability Research, 55, 19–29. doi:. 10.1111/j.1365-2788.2010.01343.x 21121991 PMC4437210

[r20] Barch D. M. (2003). Cognition in Schizophrenia: Does working memory work? Current Directions in Psychological Science, 12(4), 146–150. doi:. 10.1111/1467-8721.01251

[r21] Barch D. M. (2004). Pharmacological manipulation of human working memory. Psychopharmacology, 174, 126–135. doi:. 10.1007/s00213-003-1732-3 15205883

[r22] Barch D. M. Sheline Y. I. Csernansky J. G. Snyder A. Z. (2003). Working memory and prefrontal cortex dysfunction: Specificity to schizophrenia compared with major depression. Biological Psychiatry, 53(5), 376–384. doi:. 10.1016/S0006-3223(02)01674-8 12614990

[r23] Beier M. E. Ackerman P. L. (2005). Working memory and intelligence: Different constructs: Reply to Oberauer et al. (2005) and Kane et al. (2005). Psychological Bulletin, 131, 72-75. doi: 10.1037/0033-2909.131.1.72 15631550

[r24] Beilock S. L. Rydell R. J. McConnell A. R. (2007). Stereotype threat and working memory: Mechanisms, alleviation, and spillover. Journal of Experimental Psychology: General, 136, 256–276. doi:. 10.1037/0096-3445.136.2.256 17500650

[r25] Besson M. Schön D. Moreno S. Santos A. Magne C. (2007). Influence of musical expertise and musical training on pitch processing in music and language. Restorative Neurology and Neuroscience, 25, 399–410. 17943015

[r26] Bialystok E. (2011). Coordination of executive functions in monolingual and bilingual children. Journal of Experimental Child Psychology, 110, 461–468. doi:. 10.1016/j.jecp.2011.05.005 21683958 PMC3139691

[r27] Bialystok E. Feng X. (2009). Language proficiency and executive control in proactive interference: Evidence from monolingual and bilingual children and adults. Brain and Language, 109, 93–100. doi:. 10.1016/j.bandl.2008.09.001 18834625 PMC2699211

[r28] Biggs S. N. Bourke R. Anderson V. Jackman A. R. Killedar A. Nixon G. M. Horne R. S. C. (2011). Working memory in children with sleep-disordered breathing: Objective versus subjective measures. Sleep Medicine, 12, 887–891. doi:. 10.1016/j.sleep.2011.07.003 21924953

[r29] Bird E. K. R. Chapman R. S. (1994). Sequential recall in individuals with Down syndrome. Journal of Speech, Language, and Hearing Research: JSLHR, 37(6), 1369–1380. doi:. 10.1044/jshr.3706.1369 7877294

[r30] Blake J. Smith A. (1997). Effects of smoking and smoking deprivation on the articulatory loop of working memory. Human Psychopharmacology, 12, 259–264. 10.1002/(SICI)1099-1077(199705/06)12:3<259::AID-HUP866>3.0.CO;2-F

[r31] Bomyea J. Amir N. (2011). The effect of an executive functioning training program on working memory capacity and intrusive thoughts. Cognitive Therapy and Research, 35, 529–535. doi:. 10.1007/s10608-011-9369-8 22514357 PMC3326397

[r32] Bonifacci P. Giombini L. Bellocchi S. Contento S. (2011). Speed of processing, anticipation, inhibition and working memory in bilinguals. Developmental Science, 14, 256–269. doi:. 10.1111/j.1467-7687.2010.00974.x 22213899

[r33] Bopp K. L. Verhaeghen P. (2007). Age-related differences in control processes in verbal and visuospatial working memory: Storage, transformation, supervision, and coordination. The Journals of Gerontology: Series B, Psychological Sciences and Social Sciences, 62B, P239–P246. 10.1093/geronb/62.5.P239 17906164

[r34] Borella E. Carretti B. De Beni R. (2008). Working memory and inhibition across the adult life-span. Acta Psychologica, 128, 33–44. doi:. 10.1016/j.actpsy.2007.09.008 17983608

[r35] Borella E. Carretti B. Riboldi F. De Beni R. (2010). Working memory training in older adults: Evidence of transfer and maintenance effects. Psychology and Aging, 25, 767–778. doi:. 10.1037/a0020683 20973604

[r36] Brahmbhatt S. B. (2006). Neural correlates of verbal and nonverbal working memory deficits in individuals with schizophrenia and their high-risk siblings. Schizophrenia Research, 87, 191–204. doi:. 10.1016/j.schres.2006.05.019 16842976

[r37] Brehmer Y. Westerberg H. Bäckman L. (2012). Working-memory training in younger and older adults: Training gains, transfer, and maintenance. Frontiers in Human Neuroscience, 6, 63. 10.3389/fnhum.2012.00063 22470330 PMC3313479

[r38] Brose A. Lövdén M. Schmiedek F. (2014). Fluctuations in positive affect positively co-vary with working memory performance. Emotion, 14(1), 1–6. doi:. 10.1037/a0035210 24364855

[r39] Brose A. Schmiedek F. Lövdén M. Lindenberger U. (2012). Daily variability in working memory is coupled with negative affect: The role of attention and motivation. Emotion, 12, 605–617. doi:. 10.1037/a0024436 21787075

[r40] Brunoni A. R. Vanderhasselt M.-A. (2014). Working memory improvement with non-invasive stimulation of the dorsolateral prefrontal cortex: A systematic review and meta-analysis. Brain and Cognition, 86, 1–9. doi:. 10.1016/j.bandc.2014.01.008 24514153

[r41] Buschkuehl M. Jaeggi S. M. Hutchison S. Perrig-Chiello P. Dapp C. Muller M. Perrig W. J. (2008). Impact of working memory training on memory performance in old-old adults. Psychology and Aging, 23, 743–753. doi:. 10.1037/a0014342 19140646

[r42] Callicott J. H. Egan M. F. Mattay V. S. Bertolino A. Bone A. D. Verchinksi B. Weinberger D. R. (2003). Abnormal fMRI response of the dorsolateral prefrontal cortex in cognitively intact siblings of patients with schizophrenia. The American Journal of Psychiatry, 160, 709–719. doi:. 10.1176/appi.ajp.160.4.709 12668360

[r43] Casement M. D. Broussard J. L. Mullington J. M. Press D. Z. (2006). The contribution of sleep to improvements in working memory scanning speed: A study of prolonged sleep restriction. Biological Psychology, 72, 208–212. doi:. 10.1016/j.biopsycho.2005.11.002 16384630 PMC4120641

[r44] Cellini N. de Zambotti M. Covassin N. Sarlo M. Stegagno L. (2014). Working memory impairment and cardiovascular hyperarousal in young primary insomniacs. Psychophysiology, 51, 206–214. doi:. 10.1111/psyp.12167 24571027

[r45] Chambers R. Lo B. C. Y. Allen N. B. (2008). The impact of intensive mindfulness training on attentional control, cognitive style, and affect. Cognitive Therapy and Research, 32, 303–322. doi:. 10.1007/s10608-007-9119-0

[r46] Chavanon M.-L. Wacker J. Leue A. Stemmler G. (2007). Evidence for a dopaminergic link between working memory and agentic extraversion: An analysis of load-related changes in EEG alpha 1 activity. Biological Psychology, 74, 46–59. doi:. 10.1016/j.biopsycho.2006.07.001 16904812

[r47] Chee M. W. L. Chuah L. Y. M. Venkatraman V. Chan W. Y. Philip P. Dinges D. F. (2006). Functional imaging of working memory following normal sleep and after 24 and 35 h of sleep deprivation: Correlations of fronto-parietal activation with performance. NeuroImage, 31, 419–428. doi:. 10.1016/j.neuroimage.2005.12.001 16427321

[r48] Chiappe P. Hasher L. Siegel L. S. (2000). Working memory, inhibitory control, and reading disability. Memory & Cognition, 28, 8–17. doi:. 10.3758/BF03211570 10714133

[r49] Choo W.-C. Lee W.-W. Venkatraman V. Sheu F.-S. Chee M. W. L. (2005). Dissociation of cortical regions modulated by both working memory load and sleep deprivation and by sleep deprivation alone. NeuroImage, 25, 579–587. doi:. 10.1016/j.neuroimage.2004.11.029 15784437

[r50] Chooi W.-T. Thompson L. A. (2012). Working memory training does not improve intelligence in healthy young adults. Intelligence, 40, 531–542. doi:. 10.1016/j.intell.2012.07.004

[r51] Clarkson-Smith L. Hartley A. A. (1989). Relationships between physical exercise and cognitive abilities in older adults. Psychology and Aging, 4, 183–189. doi:. 10.1037/0882-7974.4.2.183 2789745

[r52] Colom R. Rebollo I. Palacios A. Juan-Espinosa M. Kyllonen P. C. (2004). Working memory is (almost) perfectly predicted by g. Intelligence, 32, 277–296. doi:. 10.1016/j.intell.2003.12.002

[r53] Colzato L. S. Huizinga M. Hommel B. (2009). Recreational cocaine polydrug use impairs cognitive flexibility but not working memory. Psychopharmacology, 207, 225–234. doi:. 10.1007/s00213-009-1650-0 19727676 PMC2770634

[r54] Conway A. R. A. Cowan N. Bunting M. F. Therriault D. J. Minkoff S. R. B. (2002). A latent variable analysis of working memory capacity, short-term memory capacity, processing speed, and general fluid intelligence. Intelligence, 30, 163–183. doi:. 10.1016/S0160-2896(01)00096-4

[r55] Conway R. A. Kane M. J. Engle R. W. (2003). Working memory capacity and its relation to general intelligence. Trends in Cognitive Sciences, 7(12), 547–552. doi:. 10.1016/j.tics.2003.10.005 14643371

[r56] Coolidge F. L. Segal D. L. Applequist K. (2009). Working memory deficits in personality disorder traits: A preliminary investigation in a nonclinical sample. Journal of Research in Personality, 43, 355–361. doi:. 10.1016/j.jrp.2008.12.039

[r57] Costa, P. T., & McCrea, R. R. (1978). Objective personality assessment. In M. Storandt, I. C. Siegler, & M. F. Elias (Eds.), *The clinical psychology of aging*. New York, NY, USA: Plenum Press.

[r58] Cowan, N. (1999). An embedded-processes model of working memory. In A Miyake & P. Shah (Eds.), *Models of working memory: Mechanisms of active maintenance and executive control* (pp. 62-101). Cambridge, United Kingdom: Cambridge University Press.

[r59] Cowan N. (2017). The many faces of working memory and short-term storage. Psychonomic Bulletin & Review, 24(4), 1158–1170. doi:. 10.3758/s13423-016-1191-6 27896630

[r60] Cowan N. Naveh-Benjamin M. Kilb A. Saults J. S. (2006). Life-span development of visual working memory: When is feature binding difficult? Developmental Psychology, 42, 1089–1102. doi:. 10.1037/0012-1649.42.6.1089 17087544 PMC1635970

[r61] Coy B. O’Brien W. H. Tabaczynski T. Northern J. Carels R. (2011). Associations between evaluation anxiety, cognitive interference and performance on working memory tasks. Applied Cognitive Psychology, 25, 823–832. doi:. 10.1002/acp.1765

[r62] Craik F. I. M. Bialystok E. (2006). Cognition through the lifespan: Mechanisms of change. Trends in Cognitive Sciences, 10, 131–138. doi:. 10.1016/j.tics.2006.01.007 16460992

[r63] Crichton G. E. Murphy K. J. Howe P. R. C. Buckley J. D. Bryan J. (2012). Dairy consumption and working memory performance in overweight and obese adults. Appetite, 59, 34–40. doi:. 10.1016/j.appet.2012.03.019 22459311

[r64] Cui J. Gao D. Chen Y. Zou X. Wang Y. (2010). Working memory in early-school-age children with Asperger’s syndrome. Journal of Autism and Developmental Disorders, 40, 958–967. doi:. 10.1007/s10803-010-0943-9 20108031

[r65] Dahlin E. Nyberg L. Bäckman L. Neely A. S. (2008). Plasticity of executive functioning in young and older adults: Immediate training gains, transfer, and long-term maintenance. Psychology and Aging, 23, 720–730. doi:. 10.1037/a0014296 19140643

[r66] Daneman M. Carpenter P. A. (1980). Individual differences in working memory and reading. Journal of Verbal Learning and Verbal Behavior, 19, 450–466. doi:. 10.1016/S0022-5371(80)90312-6

[r67] Datta A. Bansal V. Diaz J. Patel J. Reato D. Bikson M. (2009). Gyri-precise head model of transcranial direct current stimulation: Improved spatial focality using a ring electrode versus conventional rectangular pad. Brain Stimulation, 2(4), 201–207. 10.1016/j.brs.2009.03.005 20648973 PMC2790295

[r68] de Ribaupierre A. Lecerf T. (2006). Relationships between working memory and intelligence: Convergent evidence from a neo-Piagetian and a psychometric approach. European Journal of Cognitive Psychology, 18, 109–137. 10.1080/09541440500216127

[r69] Deluca J. Christodoulou C. Diamond B. J. Rosenstein E. D. Kramer N. Natelson B. H. (2004). Working memory deficits in chronic fatigue syndrome: Differentiating between speed and accuracy of information processing. Journal of the International Neuropsychological Society*,* 10, 101-109. doi: 10.1017/S1355617704101124 14751012

[r70] Derakshan N. Eysenck M. W. (2009). Anxiety, processing efficiency, and cognitive performance: New developments from attentional control theory. European Psychologist, 14(2), 168–176. doi:. 10.1027/1016-9040.14.2.168

[r71] D’Esposito M. Detre J. A. Alsop D. C. Shin R. K. Atlas S. Grossman M. (1995). The neural basis of the central executive system of working memory. Nature, 378, 279–281. doi:. 10.1038/378279a0 7477346

[r72] D’Esposito M. Postle B. R. Rypma B. (2000). Prefrontal cortical contributions to working memory: Evidence from event-related fMRI studies. Experimental Brain Research, 133(1), 3–11. doi:. 10.1007/s002210000395 10933205

[r73] Dietrich A. Sparling P. B. (2004). Endurance exercise selectively impairs prefrontal-dependent cognition. Brain and Cognition, 55, 516–524. doi:. 10.1016/j.bandc.2004.03.002 15223198

[r74] Dowson J. H. McLean A. Bazanis E. Toone B. Young S. Robbins T. W. Sahakian B. J. (2004). Impaired spatial working memory in adults with attention deficit/hyperactivity disorder: Comparisons with performance in adults with borderline personality disorder and in control subjects. Acta Psychiatrica Scandinavica, 110, 45–54. doi:. 10.1111/j.1600-0447.2004.00292.x 15180779

[r75] Drollette E. S. Shishido T. Pontifex M. B. Hillman C. H. (2012). Maintenance of cognitive control during and after walking in preadolescent children. Medicine and Science in Sports and Exercise, 44(10), 2017–2024. doi:. 10.1249/MSS.0b013e318258bcd5 22525770

[r76] Duff S. J. Hampson E. (2001). A sex difference on a novel spatial working memory task in humans. Brain and Cognition, 47, 470–493. doi:. 10.1006/brcg.2001.1326 11748902

[r77] Elliman N. A. Green M. W. Rogers P. J. Finch G. M. (1997). Processing-efficiency theory and the working-memory system: Impairments associated with sub-clinical anxiety. Personality and Individual Differences, 23, 31–35. doi:. 10.1016/S0191-8869(97)00016-0

[r78] Ellis, N. (2001). Memory for language. In P. Robinson (Ed.), *Cognition and second language instruction* (pp. 33-68). New York, NY, USA: Cambridge University Press.

[r79] Engle R. W. Tuholski S. W. Laughlin J. E. Conway A. R. A. (1999). Working memory, short-term memory, and general fluid intelligence: A latent-variable approach. Journal of Experimental Psychology: General, 128, 309–331. doi:. 10.1037/0096-3445.128.3.309 10513398

[r80] Ernst M. Heishman S. J. Spurgeon L. London E. D. (2001). Smoking history and nicotine effects on cognitive performance. Neuropsychopharmacology, 25, 313–319. doi:. 10.1016/S0893-133X(01)00257-3 11522460

[r81] Eysenck M. W. Derakshan N. Santos R. Calvo M. G. (2007). Anxiety and cognitive performance: Attentional control theory. Emotion, 7, 336–353. doi:. 10.1037/1528-3542.7.2.336 17516812

[r82] Fastenau P. S. Denburg N. L. Abeles N. (1996). Age differences in retrieval: Further support for the resource-reduction hypothesis. Psychology and Aging, 11, 140–146. doi:. 10.1037/0882-7974.11.1.140 8726379

[r83] Fenn K. M. Hambrick D. Z. (2012). Individual differences in working memory capacity predict sleep-dependent memory consolidation. Journal of Experimental Psychology: General, 141(3), 404–410. doi:. 10.1037/a0025268 21910555

[r84] Fink A. Grabner R. H. Neuper C. Neubauer A. C. (2005). Extraversion and cortical activation during memory performance. International Journal of Psychophysiology, 56, 129–141. doi:. 10.1016/j.ijpsycho.2004.11.002 15804448

[r85] Fisk J. E. Montgomery C. Hadjiefthyvoulou F. (2011). Visuospatial working memory impairment in current and previous ecstasy/polydrug users. Human Psychopharmacology, 26, 313–321. doi:. 10.1002/hup.1207 22700465

[r86] Fisk J. E. Warr P. (1996). Age and working memory: The role of perceptual speed, the central executive, and the phonological loop. Psychology and Aging, 11, 316–323. doi:. 10.1037/0882-7974.11.2.316 8795060

[r87] Forbes C. E. Schmader T. (2010). Retraining attitudes and stereotypes to affect motivation and cognitive capacity under stereotype threat. Journal of Personality and Social Psychology, 99, 740–754. doi:. 10.1037/a0020971 20822288 PMC2976624

[r88] Franklin M. S. Moore K. S. Yip C.-Y. Jonides J. Rattray K. Moher J. (2008). The effects of musical training on verbal memory. Psychology of Music, 36(3), 353–365. doi:. 10.1177/0305735607086044

[r89] Fredholm B. B. Bättig K. Holmén J. Nehlig A. Zvartau E. E. (1999). Actions of caffeine in the brain with special reference to factors that contribute to its widespread use. Pharmacological Reviews, 51(1), 83–133. 10049999

[r90] Fujioka T. Ross B. Kakigi R. Pantev C. Trainor L. (2006). One year of musical training affects development of auditory cortical-evoked fields in young children. Brain, 129, 2593–2608. doi:. 10.1093/brain/awl247 16959812

[r91] Gagnon L. G. Belleville S. (2011). Working memory in mild cognitive impairment and Alzheimer’s Disease: Contribution of forgetting and predictive value of complex span tasks. Neuropsychology, 25, 226–236. doi:. 10.1037/a0020919 21090897

[r92] Gaillard V. Barrouillet P. Jarrold C. Camos V. (2011). Developmental differences in working memory: Where do they come from? Journal of Experimental Child Psychology, 110, 469–479. doi:. 10.1016/j.jecp.2011.05.004 21664622

[r93] Gailliot M. T. Baumeister R. F. DeWall C. N. Maner J. K. Plant E. A. Tice D. M. Schmeichel B. J. (2007). Self-control relies on glucose as a limited energy source: Willpower is more than a metaphor. Journal of Personality and Social Psychology, 92, 325–336. doi:. 10.1037/0022-3514.92.2.325 17279852

[r94] Galletly C. A. McFarlane A. C. Clark R. (2008). Differentiating cortical patterns of cognitive dysfunction in schizophrenia and posttraumatic stress disorder. Psychiatry Research, 159, 196–206. doi:. 10.1016/j.psychres.2007.04.001 18423610

[r95] Gathercole S. E. Pickering S. J. Ambridge B. Wearing H. (2004). The structure of working memory from 4 to 15 years of age. Developmental Psychology, 40, 177–190. doi:. 10.1037/0012-1649.40.2.177 14979759

[r96] George E. M. Coch D. (2011). Music training and working memory: An ERP study. Neuropsychologia, 49, 1083–1094. doi:. 10.1016/j.neuropsychologia.2011.02.001 21315092

[r97] Gevins A. McEvoy L. K. Smith M. E. Chan C. S. Sam-Vargas L. Baum C. Ilan A. B. (2012). Long-term and within-day variability of working memory performance and EEG in individuals. Clinical Neurophysiology, 123, 1291–1299. doi:. 10.1016/j.clinph.2011.11.004 22154302 PMC3325329

[r98] Gevins A. Smith M. E. McEvoy L. K. (2002). Tracking the cognitive pharmacodynamics of psychoactive substances with combinations of behavioral and neurophysiological measures. Neuropsychopharmacology, 26, 27–39. doi:. 10.1016/S0893-133X(01)00300-1 11751030

[r99] Gilbert A. M. Fiez J. A. (2004). Integrating rewards and cognition in the frontal cortex. Cognitive, Affective & Behavioral Neuroscience, 4, 540–552. doi:. 10.3758/CABN.4.4.540 15849896

[r100] Gilbert B. Belleville S. Bherer L. Chouinard S. (2005). Study of verbal working memory in patients with Parkinson’s Disease. Neuropsychology, 19, 106–114. doi:. 10.1037/0894-4105.19.1.106 15656768

[r101] Giles G. E. Mahoney C. R. Brunye T. T. Gardony A. L. Taylor H. A. Kanarek R. B. (2012). Differential cognitive effects of energy drink ingredients: Caffeine, taurine, and glucose. Pharmacology, Biochemistry, and Behavior, 102, 569–577. doi:. 10.1016/j.pbb.2012.07.004 22819803

[r102] Gohier B. Ferracci L. Surguladze S. A. Lawrence E. Hage W. E. Kefi M. Z. Le Gall D. (2009). Cognitive inhibition and working memory in unipolar depression. Journal of Affective Disorders, 116, 100–105. doi:. 10.1016/j.jad.2008.10.028 19042027

[r103] Goldstein J. M. Jerram M. Poldrack R. Anagnoson R. Breiter H. C. Makris N. Seidman L. J. (2005). Sex differences in prefrontal cortical brain activity during fMRI of auditory verbal working memory. Neuropsychology, 19, 509–519. doi:. 10.1037/0894-4105.19.4.509 16060826

[r104] Goldstein K. E. Hazlett E. A. Savage K. R. Berline H. A. Hamilton H. K. Zelmanova Y. New A. S. (2011). Dorso- and ventro-lateral prefrontal volume and spatial working memory in schizotypal personality disorder. Behavioural Brain Research, 218, 335–340. doi:. 10.1016/j.bbr.2010.11.042 21115066 PMC3049905

[r105] Gradisar M. Terrill G. Johnston A. Douglas P. (2008). Adolescent sleep and working memory performance. Sleep and Biological Rhythms, 6, 146–154. doi:. 10.1111/j.1479-8425.2008.00353.x

[r106] Grady C. L. McIntosh A. R. Bookstein F. Horwitz B. Rapoport S. I. Haxby J. V. (1998). Age-related changes in regional cerebral blood flow during working memory for faces. NeuroImage, 8, 409–425. doi:. 10.1006/nimg.1998.0376 9811558

[r107] Grady C. L. McIntosh A. R. Horwitz B. Maisog J. M. Ungerleider L. G. Mentis M. J. Haxby J. V. (1995). Age-related reductions in human recognition memory due to impaired encoding. Science, 269, 218–221. doi:. 10.1126/science.7618082 7618082

[r108] Gray J. R. (2001). Emotional modulation of cognitive control: Approach–withdrawal states double-dissociate spatial from verbal two-back task performance. Journal of Experimental Psychology: General, 130, 436–452. doi:. 10.1037/0096-3445.130.3.436 11561919

[r109] Gray J. R. (2004). Integration of emotion and cognitive control. Current Directions in Psychological Science, 13, 46–48. doi:. 10.1111/j.0963-7214.2004.00272.x

[r110] Green M. W. Rogers P. J. (1995). Impaired cognitive function during spontaneous dieting. Psychological Medicine, 25, 1003–1010. doi:. 10.1017/S0033291700037491 8587997

[r111] Green M. W. Jones A. D. Smith I. D. Cobain M. R. Williams J. M. G. Healy H. Durlach P. J. (2003). Impairments in working memory associated with naturalistic dieting in women: No relationship between task performance and urinary 5-HIAA levels. Appetite, 40, 145–153. doi:. 10.1016/S0195-6663(02)00137-X 12781164

[r112] Greenstein J. E. Kassel J. D. (2009). The effects of smoking and smoking abstinence on verbal and visuospatial working memory capacity. Experimental and Clinical Psychopharmacology, 17, 78–90. doi:. 10.1037/a0015699 19331484

[r113] Haavisto M. L. Lehto J. E. (2005). Fluid/spatial and crystallized intelligence in relation to domain-specific working memory: A latent-variable approach. Learning and Individual Differences, 15, 1–21. doi:. 10.1016/j.lindif.2004.04.002

[r114] Hagenhoff M. Franzen N. Koppe G. Baer N. Scheibel N. Sammer G. Lis S. (2013). Executive functions in borderline personality disorder. Psychiatry Research, 210, 224–231. doi:. 10.1016/j.psychres.2013.05.016 23764434

[r115] Hale S. Rose N. S. Myerson J. Strube M. J. Sommers M. Tye-Murray N. Spehar B. (2011). The structure of working memory abilities across the adult life span. Psychology and Aging, 26, 92–110. doi:. 10.1037/a0021483 21299306 PMC3062735

[r116] Harkins S. G. (2006). Mere effort as the mediator of the evaluation-performance relationship. Journal of Personality and Social Psychology, 91, 436–455. doi:. 10.1037/0022-3514.91.3.436 16938029

[r117] Harrington M. Sawyer M. (1992). L2 working memory capacity and L2 reading skill. Studies in Second Language Acquisition, 14, 25–38. doi:. 10.1017/S0272263100010457

[r118] Hartley A. A. Speer N. K. Jonides J. Reuter-Lorenz P. A. Smith E. E. (2001). Is the dissociability of working memory systems for name identity, visual-object identity, and spatial location maintained in old age? Neuropsychology, 15, 3-17. doi:. 10.1037/0894-4105.15.1.3 11216886

[r119] Hedden T. Park D. (2001). Aging and interference in verbal working memory. Psychology and Aging, 16, 666–681. doi:. 10.1037/0882-7974.16.4.666 11766920

[r120] Hogan C. L. Mata J. Carstensen L. L. (2013). Exercise holds immediate benefits for affect and cognition in younger and older adults. Psychology and Aging, 28, 587–594. doi:. 10.1037/a0032634 23795769 PMC3768113

[r121] Holmes J. Gathercole S. E. Dunning D. L. (2009). Adaptive training leads to sustained enhancement of poor working memory in children. Developmental Science, 12, F9–F15. doi:. 10.1111/j.1467-7687.2009.00848.x 19635074

[r122] Huntley J. D. Howard R. J. (2010). Working memory in early Alzheimer’s disease: A neuropsychological review. International Journal of Geriatric Psychiatry, 25, 121–132. 10.1002/gps.2314 19672843

[r123] Ilan A. B. Smith M. E. Gevins A. (2004). Effects of marijuana on neurophysiological signals of working and episodic memory. Psychopharmacology, 176, 214–222. doi:. 10.1007/s00213-004-1868-9 15502936 PMC1463999

[r124] Ilkowska, M., & Engle, R. W. (2010). Trait and state differences in working memory capacity. In A. Gruszka, G. Matthews, & B. Szymura (Eds.), *Handbook of individual differences in cognition* (pp. 295-320). New York, NY, USA: Springer.

[r125] Jacobsen L. K. Krystal J. H. Mencl E. Westerveld M. Frost S. J. Pugh K. R. (2005). Effects of smoking and smoking abstinence on cognition in adolescent tobacco smokers. Biological Psychiatry, 57, 56–66. doi:. 10.1016/j.biopsych.2004.10.022 15607301

[r126] Jaeggi S. M. Buschkuehl M. Jonides J. Perrig W. J. (2008). Improving fluid intelligence with training on working memory. Proceedings of the National Academy of Sciences of the United States of America, 105, 6829–6833. doi:. 10.1073/pnas.0801268105 18443283 PMC2383929

[r127] Jaeggi S. M. Buschkuehl M. Perrig W. J. Meier B. (2010). The concurrent validity of the N-back task as a working memory measure. Memory, 18, 394–412. doi:. 10.1080/09658211003702171 20408039

[r128] Jaeggi S. M. Studer-Luethi B. Buschkuehl M. Su Y.-F. Jonides J. Perrig W. J. (2010). The relationship between n-back performance and matrix reasoning—implications for training and transfer. Intelligence, 38, 625–635. doi:. 10.1016/j.intell.2010.09.001

[r129] Jamieson J. P. Harkins S. G. (2007). Mere effort and stereotype threat performance effects. Journal of Personality and Social Psychology, 93, 544–564. doi:. 10.1037/0022-3514.93.4.544 17892331

[r130] Jelinek L. Moritz S. Randjbar S. Sommerfeldt D. Puschel K. Seifert D. (2008). Does the evocation of traumatic memories confound subsequent working memory performance in posttraumatic stress disorder (PTSD)? Depression and Anxiety, 25, 175–179. doi:. 10.1002/da.20300 17354268

[r131] Jenkins L. Myerson J. Hale S. Fry A. F. (1999). Individual and developmental differences in working memory across the life span. Psychonomic Bulletin & Review, 6, 28–40. doi:. 10.3758/BF03210810 12199312

[r132] Jolles D. D. Grol M. J. Van Buchem M. A. Rombouts S. A. R. B. Crone E. A. (2010). Practice effects in the brain: Changes in cerebral activation after working memory practice depend on task demands. NeuroImage, 52, 658–668. doi:. 10.1016/j.neuroimage.2010.04.028 20399274

[r133] Jones E. K. Sunram-Lea S. I. Wesnes K. A. (2012). Acute ingestion of different macronutrients differentially enhances aspects of memory and attention in healthy young adults. Biological Psychology, 89, 477–486. doi:. 10.1016/j.biopsycho.2011.12.017 22223097

[r134] Kane M. J. Conway A. R. Miura T. K. Colflesh G. J. (2007). Working memory, attention control, and the N-back task: A question of construct validity. Journal of Experimental Psychology: Learning, Memory, and Cognition, 33, 615–622. 10.1037/0278-7393.33.3.615 17470009

[r135] Kane M. J. Hambrick D. Z. Tuholski S. W. Wilhelm O. Payne T. W. Engle R. W. (2004). The generality of working memory capacity: A latent-variable approach to verbal and visuospatial memory span and reasoning. Journal of Experimental Psychology: General, 133, 189–217. doi:. 10.1037/0096-3445.133.2.189 15149250

[r136] Kane M. J. Meier M. E. Smeekens B. A. Gross G. M. Chun C. A. Silvia P. J. Kwapil T. R. (2016). Individual differences in the executive control of attention, memory, and thought, and their associations with schizotypy. Journal of Experimental Psychology: General, 145(8), 1017–1048. doi:. 10.1037/xge0000184 27454042 PMC4965188

[r137] Kasper L. J. Alderson R. M. Hudec K. L. (2012). Moderators of working memory deficits in children with attention-deficit/hyperactivity disorder (ADHD): A meta-analytic review. Clinical Psychology Review, 32, 605–617. doi:. 10.1016/j.cpr.2012.07.001 22917740

[r138] Keenan P. A. Ezzat W. H. Ginsburg K. Moore G. J. (2001). Prefrontal cortex as the site of estrogen’s effect on cognition. Psychoneuroendocrinology, 26, 577–590. doi:. 10.1016/S0306-4530(01)00013-0 11403979

[r139] Kemps E. Newson R. (2006). Comparison of adult age differences in verbal and visuo-spatial memory: The importance of “pure” parallel and validated measures. Journal of Clinical and Experimental Neuropsychology, 28, 341–356. doi:. 10.1080/13803390490918228 16618624

[r140] Kemps E. Tiggemann M. Grigg M. (2008). Food cravings consume limited cognitive resources. Journal of Experimental Psychology: Applied, 14, 247–254. doi:. 10.1037/a0012736 18808278

[r141] Kemps E. Tiggemann M. Marshall K. (2005). Relationship between dieting to lose weight and the functioning of the central executive. Appetite, 45, 287–294. doi:. 10.1016/j.appet.2005.07.002 16126305

[r142] Kemps E. Tiggemann M. Wade T. Ben-Tovim D. Breyer R. (2006). Selective working memory deficits in anorexia nervosa. European Eating Disorders Review, 14, 97–103. doi:. 10.1002/erv.685

[r143] Kensinger E. A. Corkin S. (2003). Effect of negative emotional content on working memory and long-term memory. Emotion, 3, 378–393. doi:. 10.1037/1528-3542.3.4.378 14674830

[r144] Klingberg T. Fernell E. Olesen P. J. Johnson M. Gustafsson P. Dahlström K. Westerberg H. (2005). Computerized training of working memory in children with ADHD-a randomized, controlled trial. Journal of the American Academy of Child and Adolescent Psychiatry, 44(2), 177–186. doi:. 10.1097/00004583-200502000-00010 15689731

[r145] Klingberg T. Forssberg H. Westerberg H. (2002). Training of working memory in children with ADHD. Journal of Clinical and Experimental Neuropsychology, 24(6), 781–791. doi:. 10.1076/jcen.24.6.781.8395 12424652

[r146] Komiyama T. Sudo M. Higaki Y. Kiyonaga A. Tanaka H. Ando S. (2015). Does moderate hypoxia alter working memory and executive function during prolonged exercise? Physiology & Behavior, 139, 290–296. doi:. 10.1016/j.physbeh.2014.11.057 25460539

[r147] Koppelstaetter F. Poeppel T. D. Siedentopf C. M. Ischebeck A. Verius M. Haala I. Krause B. J. (2008). Does caffeine modulate verbal working memory processes? An fMRI study. NeuroImage, 39, 492–499. doi:. 10.1016/j.neuroimage.2007.08.037 17936643

[r148] Krawczyk D. C. Gazzaley A. D’Esposito M. (2007). Reward modulation of prefrontal and visual association cortex during an incentive working memory task. Brain Research, 1141, 168–177. doi:. 10.1016/j.brainres.2007.01.052 17320835

[r149] Kudo M. Swanson H. L. (2014). Are there advantages for additive bilinguals in working memory tasks? Learning and Individual Differences, 35, 96–102. doi:. 10.1016/j.lindif.2014.07.019

[r150] Lambek R. Shevlin M. (2011). Working memory and response inhibition in children and adolescents: Age and organization issues. Scandinavian Journal of Psychology, 52, 427–432. doi:. 10.1111/j.1467-9450.2011.00899.x 21722136

[r151] Lambourne K. Audiffren M. Tomporowski P. D. (2010). Effects of acute exercise on sensory and executive processing tasks. Medicine and Science in Sports and Exercise, 42, 1396–1402. doi:. 10.1249/MSS.0b013e3181cbee11 20019631

[r152] Lange K. W. Robbins T. W. Marsden C. D. James M. Owen A. M. Paul G. M. (1992). L-dopa withdrawal in Parkinson’s disease selectively impairs cognitive performance in tests sensitive to frontal lobe dysfunction. Psychopharmacology, 107, 394–404. doi:. 10.1007/BF02245167 1615139

[r153] Lazzaretti M. Morandotti N. Sala M. Isola M. Frangou S. De Vidovich G. Brambilla P. (2012). Impaired working memory and normal sustained attention in borderline personality disorder. Acta Neuropsychiatrica, 24, 349–355. doi:. 10.1111/j.1601-5215.2011.00630.x 25287177

[r154] Lee Y.-S. Lu M.-J. Ko H.-P. (2007). Effects of skill training on working memory capacity. Learning and Instruction, 17, 336–344. doi:. 10.1016/j.learninstruc.2007.02.010

[r155] Lee Swanson H. L. Orosco M. J. Lussier C. M. (2015). Growth in literacy, cognition, and working memory in English language learners. Journal of Experimental Child Psychology, 132, 155–188. doi:. 10.1016/j.jecp.2015.01.001 25731085

[r156] Lejbak L. Crossley M. Vrbancic M. (2011). A male advantage for spatial and object but not verbal working memory using the n-back task. Brain and Cognition, 76, 191–196. doi:. 10.1016/j.bandc.2010.12.002 21411205

[r157] Li S. C. Schmiedek F. Huxhold O. Rocke C. Smith J. Lindenberger U. (2008). Working memory plasticity in old age: Practice gain, transfer, and maintenance. Psychology and Aging, 23, 731–742. doi:. 10.1037/a0014343 19140644

[r158] Lieberman H. R. Tharion W. J. Shukitt-Hale B. Speckman K. L. Tulley R. (2002). Effects of caffeine, sleep loss, and stress on cognitive performance and mood during U.S. Navy SEAL training. Psychopharmacology, 164, 250–261. doi:. 10.1007/s00213-002-1217-9 12424548

[r159] Lieberman M. D. (2000). Introversion and working memory: Central executive differences. Personality and Individual Differences, 28, 479–486. doi:. 10.1016/S0191-8869(99)00113-0

[r160] Lieberman M. D. Rosenthal R. (2001). Why introverts can’t always tell who likes them: Multitasking and nonverbal decoding. Journal of Personality and Social Psychology, 80, 294–310. doi:. 10.1037/0022-3514.80.2.294 11220447

[r161] Logie R. H. (1999). Working memory. The Psychologist, 12(4), 174–178.

[r162] Longo C. A. Kerr E. N. Smith M. L. (2013). Executive functioning in children with intractable frontal lobe or temporal lobe epilepsy. Epilepsy & Behavior, 26, 102–108. doi:. 10.1016/j.yebeh.2012.11.003 23246148

[r163] Luciana M. Collins P. F. (1997). Dopaminergic modulation of working memory for spatial but not object cues in normal humans. Journal of Cognitive Neuroscience, 9, 330–347. doi:. 10.1162/jocn.1997.9.3.330 23965011

[r164] Luo L. Craik F. I. M. Moreno S. Bialystok E. (2013). Bilingualism interacts with domain in a working memory task: Evidence from aging. Psychology and Aging, 28, 28–34. doi:. 10.1037/a0030875 23276212

[r165] Mackey, A., Philp, J., Egi, T., Fujii, A., & Tatsumi, T. (2002). Individual differences in working memory, noticing interactional feedback and L2 development. In P. Robinson (Ed.), *Individual differences and instructed language learning* (pp. 181-209). Philadelphia, PA, USA: Benjamins.

[r166] Maehler C. Schuchart K. (2009). Working memory functioning in children with learning disabilities: Does intelligence make a difference? Journal of Intellectual Disability Research, 53, 3–10. doi:. 10.1111/j.1365-2788.2008.01105.x 19093981

[r167] Mahoney C. R. Castellani J. Kramer M. Young A. Lieberman H. R. (2007). Tyrosine supplementation mitigates working memory decrements during cold exposure. Physiology & Behavior, 92, 575–582. doi:. 10.1016/j.physbeh.2007.05.003 17585971

[r168] Malapalani C. Pillon B. Dubois B. Agid Y. (1994). Impaired simultaneous cognitive task performance in Parkinson’s disease: A dopamine-related dysfunction. Neurology*,* 44, 319–326. doi: 10.1212/WNL.44.2.319 8309583

[r169] Mammarella N. Cornoldi C. Donadello E. (2003). Visual but not spatial working memory deficit in children with spina bifida. Brain and Cognition, 53, 311–314. doi:. 10.1016/S0278-2626(03)00132-5 14607170

[r170] Martínez K. Colom R. (2009). Working memory capacity and processing efficiency predict fluid but not crystallized and spatial intelligence: Evidence supporting the neural noise hypothesis. Personality and Individual Differences, 46, 281–286. doi:. 10.1016/j.paid.2008.10.012

[r171] Martins A. Q. Kavussanu M. Willoughby A. Ring C. (2013). Moderate intensity exercise facilitates working memory. Psychology of Sport and Exercise, 14, 323–328. doi:. 10.1016/j.psychsport.2012.11.010

[r172] Martinussen R. Hayden J. Hogg-Johnsen S. Tannock R. (2005). A meta-analysis of working memory impairments in children with attention/deficit/hyperactivity disorder. Journal of the American Academy of Child and Adolescent Psychiatry, 44, 377–384. doi:. 10.1097/01.chi.0000153228.72591.73 15782085

[r173] Mathias J. L. Kent P. S. (1998). Neuropsychological consequences of extreme weight loss and dietary restriction in patients with anorexia nervosa. Journal of Clinical and Experimental Neuropsychology, 20, 548–564. 10.1076/jcen.20.4.548.1476 9892058

[r174] McDowell S. Whyte J. D’Esposito M. (1997). Working memory impairments in traumatic brain injury: Evidence from a dual-task paradigm. Neuropsychologia, 35, 1341–1353. doi:. 10.1016/S0028-3932(97)00082-1 9347480

[r175] McGinnis D. Zelinski E. M. (2003). Understanding unfamiliar words in young, young-old, and old-old adults: Inferential processing and the abstraction-deficit hypothesis. Psychology and Aging, 18, 497–509. doi:. 10.1037/0882-7974.18.3.497 14518811

[r176] Melby-Lervåg M. Hulme C. (2013). Is working memory training effective? A meta-analytic review. Developmental Psychology, 49, 270–291. doi:. 10.1037/a0028228 22612437

[r177] Messier C. (2004). Glucose improvement of memory: A review. European Journal of Pharmacology, 490(1-3), 33–57. doi:. 10.1016/j.ejphar.2004.02.043 15094072

[r178] Mezzacappa E. Buckner J. C. (2010). Working memory training for children with attention problems or hyperactivity: A school-based pilot study. School Mental Health, 2(4), 202–208. doi:. 10.1007/s12310-010-9030-9

[r179] Mikels J. A. Larkin G. R. Reuter-Lorenz P. A. Carstensen L. L. (2005). Divergent trajectories in the aging mind: Changes in working memory for affective versus visual information with age. Psychology and Aging, 20, 542–553. doi:. 10.1037/0882-7974.20.4.542 16420130 PMC2746384

[r180] Miyake A. (2001). Individual differences in working memory: Introduction to the special section. Journal of Experimental Psychology: General, 130, 163–168. doi:. 10.1037/0096-3445.130.2.163 11409096

[r181] Miyake, A., & Friedman, N. P. (1998). Individual differences in second language proficiency: Working memory as language aptitude. In A. Healy & L. Bourne (Eds.), *Foreign language learning* (pp. 339-364). Mahwah, NJ, USA: Erlbaum.

[r182] Morales J. Calvo A. Bialystok E. (2013). Working memory development in monolingual and bilingual children. Journal of Experimental Child Psychology, 114, 187–202. doi:. 10.1016/j.jecp.2012.09.002 23059128 PMC3508395

[r183] Müller U. Steffenhagen N. Regenthal R. Bublak P. (2004). Effects of modafinil on working memory processes in humans. Psychopharmacology, 177, 161–169. doi:. 10.1007/s00213-004-1926-3 15221200

[r184] Muller U. von Cramon Y. Pollmann S. (1998). D1- versus D2-receptor modulation of visuospatial working memory in humans. The Journal of Neuroscience, 18, 2720–2728. 9502829 10.1523/JNEUROSCI.18-07-02720.1998 PMC6793089

[r185] Munir F. Cornish K. M. Wilding J. (2000). Nature of the working memory deficit in fragile-X syndrome. Brain and Cognition, 44, 387–401. doi:. 10.1006/brcg.1999.1200 11104532

[r186] Myerson J. Hale S. Rhee S. H. Jenkins L. (1999). Selective interference with verbal and spatial working memory in young and older adults. The Journals of Gerontology: Series B, Psychological Sciences and Social Sciences, 54B, P161–P164. doi:. 10.1093/geronb/54B.3.P161 10363037

[r187] Nagel B. J. Ohannessian A. Cummins K. (2007). Performance dissociation during verbal and spatial working memory tasks. Perceptual and Motor Skills, 105, 243–250. doi:. 10.2466/pms.105.1.243-250 17918571

[r188] Nitsche M. A. Liebetanz D. Antal A. Lang N. Tergau F. Paulus W. (2003). Modulation of cortical excitability by weak direct current stimulation – technical, safety and functional aspects. Supplements to Clinical Neurophysiology, 56, 255–276. doi:. 10.1016/S1567-424X(09)70230-2 14677403

[r189] Nitsche M. A. Paulus W. (2000). Excitability changes induced in the human motor cortex by weak transcranial direct current stimulation. The Journal of Physiology, 527(3), 633–639. doi:. 10.1111/j.1469-7793.2000.t01-1-00633.x 10990547 PMC2270099

[r190] Nouchi R. Taki Y. Takeuchi H. Sekiguchi A. Hashizume H. Nozawa T. Kawashima R. (2014). Four week of combination exercise training improved executive functions, episodic memory, and processing speed in healthy elderly people: Evidence from a randomized controlled trial. Age, 36, 787–799. doi:. 10.1007/s11357-013-9588-x 24065294 PMC4039261

[r191] Numminen H. Service E. Ahonen T. Ruoppila I. (2001). Working memory and everyday cognition in adults with Down’s syndrome. Journal of Intellectual Disability Research, 45, 157–168. doi:. 10.1046/j.1365-2788.2001.00298.x 11298256

[r192] O’Brien C. Mahoney C. Tharion W. J. Sils I. V. Castellani J. W. (2007). Dietary tyrosine benefits cognitive and psychomotor performance during body cooling. Physiology & Behavior, 90, 301–307. doi:. 10.1016/j.physbeh.2006.09.027 17078981

[r193] O’Hearn K. Courtney S. Street W. Landau B. (2009). Working memory impairment in people with Williams syndrome: Effects of delay, task, and stimuli. Brain and Cognition, 69, 495–503. doi:. 10.1016/j.bandc.2008.10.004 19084315 PMC2745717

[r194] Ornstein P. A. Schaaf J. M. Hooper S. R. Hatton D. D. Mirrett P. Bailey D. B. (2008). Memory skills of boys with fragile X syndrome. American Journal of Mental Retardation, 113, 453–465. doi:. 10.1352/2008.113:453-465 19127656

[r195] Orsini A. Simoetta S. Marmorato M. S. (2004). Corsi’s block-tapping test: Some characteristics of the spatial path which influence memory. Perceptual and Motor Skills, 98, 382–388. doi:. 10.2466/pms.98.2.382-388 15141901

[r196] Osaka M. Osaka N. Groner R. (1993). Language-independent working memory: Evidence from German and French reading span tests. Bulletin of the Psychonomic Society, 31, 117–118. doi:. 10.3758/BF03334156

[r197] Owen A. M. Iddon J. L. Hodges J. R. Summers B. A. Robbins T. W. (1997). Spatial and non-spatial working memory at different stages of Parkinson’s disease. Neuropsychologia, 35(4), 519–532. doi:. 10.1016/S0028-3932(96)00101-7 9106280

[r198] Owen L. Scholey A. B. Finnegan Y. Hu H. Sunram-Lea S. I. (2012). The effect of glucose dose and fasting interval on cognitive function: A double-blind placebo-controlled, six-way crossover study. Psychopharmacology, 220, 577–589. doi:. 10.1007/s00213-011-2510-2 21979440

[r199] Parbery-Clark A. Skoe E. Lam C. Kraus N. (2009). Musician enhancement for speech-in-noise. Ear and Hearing, 30(6), 653–661. doi:. 10.1097/AUD.0b013e3181b412e9 19734788

[r200] Park D. C. Lautenschlager G. Hedden T. Davidson N. S. Smith A. D. Smith P. K. (2002). Models of visuospatial and verbal memory across the adult life span. Psychology and Aging, 17, 299–320. doi:. 10.1037/0882-7974.17.2.299 12061414

[r201] Park S. Knopick C. McGurck S. Meltzer H. Y. (2000). Nicotine impairs spatial working memory while leaving spatial attention intact. Neuropsychopharmacology, 22, 200–209. doi:. 10.1016/S0893-133X(99)00098-6 10649832

[r202] Parmenter B. A. Shucard J. L. Benedict R. H. B. Shucard D. W. (2006). Working memory deficits in multiple sclerosis: Comparison between the n-back task and the Paced Auditory Serial Addition Test. Journal of the International Neuropsychological Society, 12, 677–687. doi:. 10.1017/S1355617706060826 16961949

[r203] Paxton J. L. Barch D. M. Racine C. A. (2008). Cognitive control, goal maintenance, and prefrontal function in healthy aging. Cerebral Cortex, 18, 1010–1028. doi:. 10.1093/cercor/bhm135 17804479 PMC2904686

[r204] Pio de Almeida L. S. Jansen K. Kohler C. A. Pinheiro R. T. da Silva R. A. Bonini J. S. (2012). Working and short-term memories are impaired in postpartum depression. Journal of Affective Disorders, 136, 1238–1242. doi:. 10.1016/j.jad.2011.09.031 22100126

[r205] Rampacher F. Lennertz L. Vogeley A. Schulze-Rauschenbach S. Kathmann N. Falkai P. Wagner M. (2010). Evidence for specific cognitive deficits in visual information processing in patients with OCD compared to patients with unipolar depression. Progress in Neuro-Psychopharmacology & Biological Psychiatry, 34, 984–991. doi:. 10.1016/j.pnpbp.2010.05.008 20472013

[r206] Rapee R. M. (1993). The utilization of working memory by worry. Behaviour Research and Therapy, 31, 617–620. doi:. 10.1016/0005-7967(93)90114-A 8347121

[r207] Reay J. L. Scholey A. B. Kennedy D. O. (2010). Panax ginseng (G115) improves aspects of working memory performance and subjective ratings of calmness in healthy young adults. Human Psychopharmacology, 25, 462–471. doi:. 10.1002/hup.1138 20737519

[r208] Redick T. S. Lindsey D. R. (2013). Complex span and n-back measures of working memory: A meta-analysis. Psychonomic Bulletin & Review, 20, 1102–1113. doi:. 10.3758/s13423-013-0453-9 23733330

[r209] Redick T. S. Broadway J. M. Meier M. E. Kuriakose P. S. Unsworth N. Kane M. J. Engle R. W. (2012). Measuring working memory capacity with automated complex span tasks. European Journal of Psychological Assessment, 28, 164–171. doi:. 10.1027/1015-5759/a000123

[r210] Richmond L. L. Morrison A. B. Chein J. M. Olson I. R. (2011). Working memory training and transfer in older adults. Psychology and Aging, 26, 813–822. doi:. 10.1037/a0023631 21707176

[r211] Robert M. Savoie N. (2006). Are there gender differences in verbal and visuospatial working-memory resources? European Journal of Cognitive Psychology, 18, 378–397. doi:. 10.1080/09541440500234104

[r212] Rose E. J. Ebmeier K. P. (2006). Pattern of impaired working memory during major depression. Journal of Affective Disorders, 90, 149–161. doi:. 10.1016/j.jad.2005.11.003 16364451

[r213] Ruocco A. C. Direkoglu E. (2013). Delineating the contributions of sustained attention and working memory to individual differences in mindfulness. Personality and Individual Differences, 54, 226–230. doi:. 10.1016/j.paid.2012.08.037

[r214] Salthouse T. A. (1994). The aging of working memory. Neuropsychology, 8, 535–543. doi:. 10.1037/0894-4105.8.4.535

[r215] Salthouse T. A. (1995). Differential age-related influences on memory for verbal-symbolic information and visual-spatial information? Journals of Gerontology: Psychological Sciences, 50B, 193–201. 10.1093/geronb/50B.4.P193 7541704

[r216] Sanada M. Ikeda K. Kimura K. Hasegawa T. (2013). Motivation enhances visual working memory capacity through the modulation of central cognitive processes. Psychophysiology, 50, 864–871. doi:. 10.1111/psyp.12077 23834356

[r217] Sawyer, M., & Ranta, L. (2001). Aptitude, individual differences, and instructional design. In P. Robinson (Ed.), *Cognition and second language instruction* (pp. 319-354). Cambridge, United Kingdom: Cambridge University Press.

[r218] Schmader T. Johns M. (2003). Converging evidence that stereotype threat reduces working memory capacity. Journal of Personality and Social Psychology, 85, 440–452. doi:. 10.1037/0022-3514.85.3.440 14498781

[r219] Schmidt H. Jogia J. Fast K. Christodoulou T. Haldane M. Kumari V. Frangou S. (2009). No gender differences in brain activation during the n-back task: An fMRI study in healthy individuals. Human Brain Mapping, 30, 3609–3615. doi:. 10.1002/hbm.20783 19387979 PMC6870785

[r220] Schmiedek F. Lövdén M. Lindenberger U. (2010). Hundred days of cognitive training enhance broad abilities in adulthood: Findings from the COGITO study. Frontiers in Aging Neuroscience, 2, 27. 10.3389/fnagi.2010.00027 20725526 PMC2914582

[r221] Scholey A. B. Harper S. Kennedy D. O. (2001). Cognitive demand and blood glucose. Physiology & Behavior, 73(4), 585–592. doi:. 10.1016/S0031-9384(01)00476-0 11495663

[r222] Scholey A. Ossoukhova A. Owen L. Ibarra A. Pipingas A. He K. Stough C. (2010). Effects of American ginseng (*Panax quinquefolius*) on neurocognitive function: An acute, randomized, double-blind, placebo-controlled, crossover study. Psychopharmacology, 212, 345–356. doi:. 10.1007/s00213-010-1964-y 20676609 PMC2952762

[r223] Schoofs D. Wolf O. T. Smeets T. (2009). Cold pressor stress impairs performance on working memory tasks requiring executive functions in healthy young men. Behavioral Neuroscience, 123, 1066–1075. doi:. 10.1037/a0016980 19824773

[r224] Schweinsburg A. D. Nagel B. J. Tapert S. F. (2005). fMRI reveals alteration of spatial working memory networks across adolescence. Journal of the International Neuropsychological Society, 11, 631–644. doi:. 10.1017/S1355617705050757 16212691 PMC2270702

[r225] Schweizer K. Moosbrugger H. (2004). Attention and working memory as predictors of intelligence. Intelligence, 32, 329–347. doi:. 10.1016/j.intell.2004.06.006

[r226] Sellaro R. Hommel B. Manai M. Colzato L. S. (2015). Preferred, but not objective temperature predicts working memory depletion. Psychological Research, 79, 282–288. doi:. 10.1007/s00426-014-0558-4 24652342

[r227] Service E. (1992). Phonology, working memory, and foreign language learning. Quarterly Journal of Experimental Psychology, 45A, 21–50. doi:. 10.1080/14640749208401314 1636010

[r228] Shackman A. J. Sarinopoulos I. Maxwell J. S. Pizzagalli D. A. Lavric A. Davidson R. J. (2006). Anxiety selectively disrupts visuospatial working memory. Emotion, 6, 40–61. doi:. 10.1037/1528-3542.6.1.40 16637749

[r229] Shaw J. Tiggemann M. (2004). Dieting and working memory: Preoccupying cognitions and the role of the articulatory control process. British Journal of Health Psychology, 9, 175–185. doi:. 10.1348/135910704773891032 15125803

[r230] Shipstead Z. Redick T. S. Engle R. W. (2012). Is working memory training effective? Psychological Bulletin, 138, 628–654. doi:. 10.1037/a0027473 22409508

[r231] Shurtleff D. Thomas J. R. Schrot J. Kowalski K. Harford R. (1994). Tyrosine reverses a cold-induced working memory deficit in humans. Pharmacology, Biochemistry, and Behavior, 47, 935–941. doi:. 10.1016/0091-3057(94)90299-2 8029265

[r232] Siegel L. S. (1994). Working memory and reading: A lifespan perspective. International Journal of Behavioral Development, 17, 109–124. doi:. 10.1177/016502549401700107

[r233] Smillie L. D. Gokcen E. (2010). Caffeine enhances working memory for extraverts. Biological Psychology, 85, 496–498. doi:. 10.1016/j.biopsycho.2010.08.012 20816912

[r234] Smith A. P. (2013). Caffeine, extraversion and working memory. Journal of Psychopharmacology, 27, 71–76. doi:. 10.1177/0269881112460111 23015541

[r235] Smith A. P. Clark R. Gallagher J. (1999). Breakfast cereal and caffeinated coffee: Effects on working memory, attention, mood, and cardiovascular function. Physiology & Behavior, 67, 9–17. doi:. 10.1016/S0031-9384(99)00025-6 10463623

[r236] Soga K. Shishido T. Nagatomi R. (2015). Executive function during and after acute moderate aerobic exercise in adolescents. Psychology of Sport and Exercise, 16, 7–17. doi:. 10.1016/j.psychsport.2014.08.010

[r237] Sommerfield A. J. Deary I. J. McAulay V. Frier B. M. (2003). Moderate hypoglycemia impairs multiple memory functions in healthy adults. Neuropsychology, 17, 125–132. doi:. 10.1037/0894-4105.17.1.125 12597081

[r238] Sorg B. A. Whitney P. (1992). The effect of trait anxiety and situational stress on working memory capacity. Journal of Research in Personality, 26, 235–241. doi:. 10.1016/0092-6566(92)90041-2

[r239] Speck O. Ernst T. Braun J. Koch C. Miller E. Chang L. (2000). Gender differences in the functional organization of the brain for working memory. Neuroreport, 11, 2581–2585. 10.1097/00001756-200008030-00046 10943726

[r240] Sprenger A. M. Atkins S. M. Bolger D. J. Harbison J. I. Novick J. M., Chrabaszcz J. Dougherty M. R. (2013). Training working memory: Limits of transfer. Intelligence, 41, 638–663. doi:. 10.1016/j.intell.2013.07.013

[r241] Steele C. M. (1997). A threat in the air: How stereotypes shape intellectual identity and performance. The American Psychologist, 52, 613–629. 10.1037/0003-066X.52.6.613 9174398

[r242] Steele S. D. Minshew N. J. Luna B. Sweeney J. A. (2007). Spatial working memory deficits in autism. Journal of Autism and Developmental Disorders, 37, 605–612. doi:. 10.1007/s10803-006-0202-2 16909311

[r243] Steenari M. R. Vuontela V. Paavonen E. J. Carlson S. Fjallberg M. Aronen E. T. (2003). Working memory and sleep in 6- to 13-year-old schoolchildren. Journal of the American Academy of Child and Adolescent Psychiatry, 42, 85–92. doi:. 10.1097/00004583-200301000-00014 12500080

[r244] Stevens A. Burkhardt M. Hautzinger M. Schwarz J. Unckel C. (2004). Borderline personality disorder: Impaired visual perception and working memory. Psychiatry Research, 125, 257–267. doi:. 10.1016/j.psychres.2003.12.011 15051186

[r245] Studer-Luethi B. Jaeggi S. M. Buschkuehl M. Perrig W. J. (2012). Influence of neuroticism and conscientiousness on working memory training outcome. Personality and Individual Differences, 53, 44–49. doi:. 10.1016/j.paid.2012.02.012

[r246] Sünram-Lea S. I. Foster J. K. Durlach P. Perez C. (2002). The effect of retrograde and anterograde glucose administration on memory performance in healthy young adults. Behavioural Brain Research, 134, 505–516. doi:. 10.1016/S0166-4328(02)00086-4 12191837

[r247] Taib M. N. M. Shariff Z. M. Wesnes K. A. Abu Saad H. Sariman S. (2012). The effect of high lactose-isomaltulose on cognitive performance of young children: A double blind cross-over design study. Appetite, 58, 81–87. doi:. 10.1016/j.appet.2011.09.004 21986189

[r248] Tiggemann M. Kemps E. Parnell J. (2010). The selective impact of chocolate craving on visuospatial working memory. Appetite, 55, 44–48. doi:. 10.1016/j.appet.2010.03.010 20307600

[r249] Tine M. Gotlieb R. (2013). Gender-, race-, and income-based stereotype threat: The effects of multiple stigmatized aspects of identity on math performance and working memory function. Social Psychology of Education, 16, 353–376. doi:. 10.1007/s11218-013-9224-8

[r250] Turner M. L. Engle R. W. (1989). Is working memory capacity task dependent? Journal of Memory and Language, 28, 127–154. 10.1016/0749-596X(89)90040-5

[r251] Unsworth N. Engle R. W. (2005). Working memory capacity and fluid abilities: Examining the correlation between Operation Span and Raven. Intelligence, 33, 67–81. doi:. 10.1016/j.intell.2004.08.003

[r252] Van der Molen M. J. Van Luit J. E. H. Jongmans M. J. Van der Molen M. W. (2007). Verbal working memory in children with mild intellectual disabilities. Journal of Intellectual Disability Research, 51, 162–169. doi:. 10.1111/j.1365-2788.2006.00863.x 17217480

[r253] Vecchi T. Richardson J. T. E. Cavallini E. (2005). Passive storage versus active processing in working memory: Evidence from age-related variations in performance. The European Journal of Cognitive Psychology, 17, 521–539. 10.1080/09541440440000140

[r254] Virués-Ortega J. Buela-Casal G. Garrido E. Alcázar B. (2004). Neuropsychological functioning associated with high-altitude exposure. Neuropsychology Review, 14, 197–224. doi:. 10.1007/s11065-004-8159-4 15796116

[r255] Visu-Petra L. Cheie L. Benga O. Alloway T. P. (2011). Effects of anxiety on memory storage and updating in young children. International Journal of Behavioral Development, 35, 38–47. doi:. 10.1177/0165025410368945

[r256] Voyer D. Voyer S. Bryden M. P. (1995). Magnitude of sex differences in spatial abilities: A meta-analysis and consideration of critical variables. Psychological Bulletin, 117, 250–270. doi:. 10.1037/0033-2909.117.2.250 7724690

[r257] Vreugdenburg L. Bryan J. Kemps E. (2003). The effect of self-initiated weight-loss dieting on working memory: The role of preoccupying cognitions. Appetite, 41, 291–300. doi:. 10.1016/S0195-6663(03)00107-7 14637328

[r258] Walter C. (2004). Transfer of reading comprehension skills to L2 is linked to mental representations of text and to L2 working memory. Applied Linguistics, 25, 315–339. doi:. 10.1093/applin/25.3.315

[r259] Wang P. P. Bellugi U. (1994). Evidence from two genetic syndromes for a dissociation between verbal and visual-spatial short-term memory. Journal of Clinical and Experimental Neuropsychology, 16(2), 317–322. doi:. 10.1080/01688639408402641 8021317

[r260] Wareing M. Fisk J. E. Murphy P. Montgomery C. (2004). Verbal working memory deficits in current and previous users of MDMA. Human Psychopharmacology, 19, 225–234. doi:. 10.1002/hup.586 15181650

[r261] Weiss M. D. Tamisier R. Boucher J. Lynch M. Gilmartin G. Weiss J. W. Thomas R. J. (2009). A pilot study of sleep, cognition, and respiration under 4 weeks of intermittent nocturnal hypoxia in adult humans. Sleep Medicine, 10, 739–745. doi:. 10.1016/j.sleep.2008.07.013 19282237 PMC3891502

[r262] Wesnes K. A. Ward T. McGinty A. Petrini O. (2000). The memory enhancing effects of a Gingko biloba/Panax ginseng combination in healthy middle-aged volunteers. Psychopharmacology, 152, 353–361. doi:. 10.1007/s002130000533 11140327

[r263] Westerberg H. Klingberg T. (2007). Changes in cortical activity after training of working memory—A single-subject analysis. Physiology & Behavior, 92, 186–192. doi:. 10.1016/j.physbeh.2007.05.041 17597168

[r264] Williamon A. Egner T. (2004). Memory structures for encoding and retrieving a piece of music: An ERP investigation. Brain Research. Cognitive Brain Research, 22, 36–44. doi:. 10.1016/j.cogbrainres.2004.05.012 15561499

[r265] Williams J. N. (1999). Memory, attention, and inductive learning. Studies in Second Language Acquisition, 21, 1–48. 10.1017/S0272263199001011

[r266] Wilson S. J. Sayette M. A. Fiez J. A. Brough E. (2007). Carry-over effects of smoking cue exposure on working memory performance. Nicotine & Tobacco Research, 9, 613–619. doi:. 10.1080/14622200701243144 17454718 PMC2626274

[r267] Wittmann M. Carter O. Hasler F. Cahn B. R. Grimburg U. Spring P. Vollenweider F. X. (2007). Effects of psilocybin on time perception and temporal control of behavior in humans. Journal of Psychopharmacology, 21, 50–64. doi:. 10.1177/0269881106065859 16714323

[r268] Yan X. Zhang J. Gong Q. (2011). Prolonged high-altitude residence impacts verbal working memory: An fMRI study. Experimental Brain Research, 208, 437–445. doi:. 10.1007/s00221-010-2494-x 21107542

[r269] Yan X. Zhang J. Shi J. Gong Q. Weng X. (2010). Cerebral and functional adaptation with chronic hypoxia exposure: A multi-modal MRI study. Brain Research, 1348, 21–29. doi:. 10.1016/j.brainres.2010.06.024 20599837

[r270] Zeidan F. Johnson S. K. Diamond B. J. David Z. Goolkasian P. (2010). Mindfulness meditation improves cognition: Evidence of brief mental training. Consciousness and Cognition, 19, 597–605. doi:. 10.1016/j.concog.2010.03.014 20363650

[r271] Zinke K. Zeintl M. Eschen A. Herzog C. Kliegel M. (2012). Potentials and limits of plasticity induced by working memory training in old-old age. Gerontology, 58, 79–87. doi:. 10.1159/000324240 21430358

[r272] Zinke K. Zeintl M. Rose N. S. Putzmann J. Pydde A. Kliegel M. (2014). Working memory training and transfer in older adults: Effects of age, baseline performance, and training gains. Developmental Psychology, 50, 304–315. doi:. 10.1037/a0032982 23688173

